# Phenotyping of Different Italian Durum Wheat Varieties in Early Growth Stage With the Addition of Pure or Digestate-Activated Biochars

**DOI:** 10.3389/fpls.2021.782072

**Published:** 2021-12-20

**Authors:** Arianna Latini, Fabio Fiorani, Patrizia Galeffi, Cristina Cantale, Annamaria Bevivino, Nicolai David Jablonowski

**Affiliations:** ^1^Italian National Agency for New Technologies, Energy and Sustainable Economic Development, ENEA Casaccia Research Center, Rome, Italy; ^2^Institute of Bio- and Geosciences, IBG-2, Plant Sciences, Forschungszentrum Jülich GmbH, Jülich, Germany

**Keywords:** biochar, digestate, *Triticum durum*, plant phenotyping, early growth stage, evapotranspiration, projected shoot area, genotype-dependence

## Abstract

This study aims to highlight the major effects of biochar incorporation into potting soil substrate on plant growth and performance in early growth stages of five elite Italian varieties of durum wheat (*Triticum durum*). The biochars used were obtained from two contrasting feedstocks, namely wood chips and wheat straw, by gasification under high temperature conditions, and were applied in a greenhouse experiment either as pure or as nutrient-activated biochar obtained by incubation with digestate. The results of the experiment showed that specific genotypes as well as different treatments with biochar have significant effects on plant response when looking at shoot traits related to growth. The evaluated genotypes could be clustered in two main distinct groups presenting, respectively, significantly increasing (Duilio, Iride, and Saragolla varieties) and decreasing (Marco Aurelio and Grecale varieties) values of projected shoot system area (PSSA), fresh weight (FW), dry weight (DW), and plant water loss by evapotranspiration (ET). All these traits were correlated with Pearson correlation coefficients ranging from 0.74 to 0.98. Concerning the treatment effect, a significant alteration of the mentioned plant traits was observed when applying biochar from wheat straw, characterized by very high electrical conductivity (EC), resulting in a reduction of 34.6% PSSA, 43.2% FW, 66.9% DW, and 36.0% ET, when compared to the control. Interestingly, the application of the same biochar after nutrient spiking with digestate determined about a 15–30% relief from the abovementioned reduction induced by the application of the sole pure wheat straw biochar. Our results reinforce the current basic knowledge available on biological soil amendments as biochar and digestate.

## Introduction

Nowadays, the application of biochar, the fine-grained charcoal rich in recalcitrant organic carbon, represents a valuable and sustainable strategy in agriculture for enhancing soil fertility and, at the same time, mitigating anthropogenic greenhouse gas emission (Lehmann, 2007). For its physicochemical and structural characteristics, biochar has direct impact on soil bulk density, water content, porosity, cation exchange capacity, and nutrient content. In particular, it can contribute to retain nutrients into soil, preventing their runoff or leaching, and increasing their availability for root uptake (Chen et al., 2019; Sakhiya et al., 2020). It has been assessed that biochar carbon and nutrient contents depend on the organic material contained in the original biomass feedstock used for its production, while biochar surface chemical properties as well as pore size depend more on the pyrolysis temperature (Lei and Zhang, 2013; Zhao et al., 2013). In addition, the effects of biochar application may vary between laboratory-scale and field-based studies and across different agroclimatic zones (Vijay et al., 2021).

Durum wheat (*Triticum durum* L. ssp. *durum* Desf.) is an economically important crop because of its unique characteristics and derived food products, pasta in particular. It provides an important source of energy, supplying a range of vitamins, minerals, and other nutritional compounds essential in the human diet (Grant et al., 2012). In the literature, several studies report contrasting effects of biochar on wheat plant growth and yield, in a different way depending on biochar type, application rate, soil, and nutrient content. Increased durum wheat yields have been reported in biochar amended fields (Baronti et al., 2010; Vaccari et al., 2011). Alburquerque et al. (2013) showed that in pot-grown durum wheat, biochar had a low effect on grain yield in nutrient-poor soil, while a 20–30% yield increase was observed when maximum mineral fertilization was applied. Biochar from fruit peels and milk tea waste improved bread wheat growth and grain yield as well as soil fertility status in a field study (Sial et al., 2019). Shahzad et al. (2019) observed that biochar application increased bread wheat grain yield, protein content, and total nitrogen uptake compared with plots with no biochar, but they also underpinned that reduced tillage was much more economically profitable than biochar application. Under greenhouse conditions, Bista et al. (2019) reported that biochar amendment at rates up to 22.4 Mg ha^–1^ increased wheat shoot and root biomass, independent of the addition of fertilizer, while a double biochar application rate determined a biomass reduction, particularly under fertilized conditions.

In sustainable agriculture, biochar addition should be planned according to a specific fertilization scheme taking into account environmental conditions, and chemical fertilization should be at least partially replaced by organic fertilization (Ayaz et al., 2021; Latini et al., 2021; Roy et al., 2021). In this regard, anaerobic digestate (AD) obtained after biogas production using plant biomass (e.g., maize) and/or manure as a feedstock has been proposed to replace inorganic fertilizer to maintain grassland productivity at less environmental cost (Walsh et al., 2012; Nkoa, 2014). Beneficial effects of digestates on plant nutrition and soil health under agricultural field conditions have been described in numerous studies recently (Doyeni et al., 2021; Grillo et al., 2021; Pastorelli et al., 2021). However, the composition, properties, and nutrient value of digestates may vary depending on their feedstock origin, e.g., manure, organic wastes, plant biomass, etc.

In this study, two types of biochar, one from wood chips and the other from wheat straw, were used to evaluate how they can affect and modify wheat plant growth. Both types of biochar have been applied pure or after nutrient spiking by incubation with a maize silage digestate. As shown in an earlier study, the use of digestate-activated biochar showed a significant increase in productivity in juvenile maize, demonstrating an improved nutrient supply (Dietrich et al., 2020). The fertilizing potential of the pure digestate and its beneficial effects on soil even on the longer term have also been shown in previous studies on maize and the perennial energy plant *Sidahermaphrodita* L. Rusby under greenhouse and outdoor conditions (Nabel et al., 2017; Robles-Aguilar et al., 2019). The current experiment was carried out in the ScreenHouse, an imaging-based phenotyping platform (IBG-2: Plant Sciences, Forschungszentrum Jülich GmbH, Jülich Germany), providing continuous, robot-assisted information on plant aboveground biomass (canopy) architecture. Overall, the behavior of five elite Italian durum wheat varieties grown under different biochar treatments has been assessed in a greenhouse phenotyping experiment designed to monitor plant growth performance during the early development and growth stages. Our aim was to get more insights into the following aspects: (i) the influence of genotype on aboveground plant growth-related properties in the different soil applied biochar amendments; (ii) the influence of the biochar feedstock on biochar chemical nutrient composition and, therefore, plant growth performance; (iii) the different short-term effects attainable by the soil application of pure or nutrient-spiked biochar through incubation with digestate on plant growth-associated traits.

## Materials and Methods

### Greenhouse Experimental Design

The greenhouse experiment was carried out in the ScreenHouse phenotyping station, in the PhyTec Experimental Greenhouse, at the Institute of Bio- and Geosciences, Plant Sciences (IBG-2), Forschungszentrum Jülich GmbH, Germany (50°54′36″N, 6°24′49″E). This phenotyping platform has been already well-described by Nakhforoosh et al. (2016) and Scharr et al. (2017).

**FIGURE 1:**
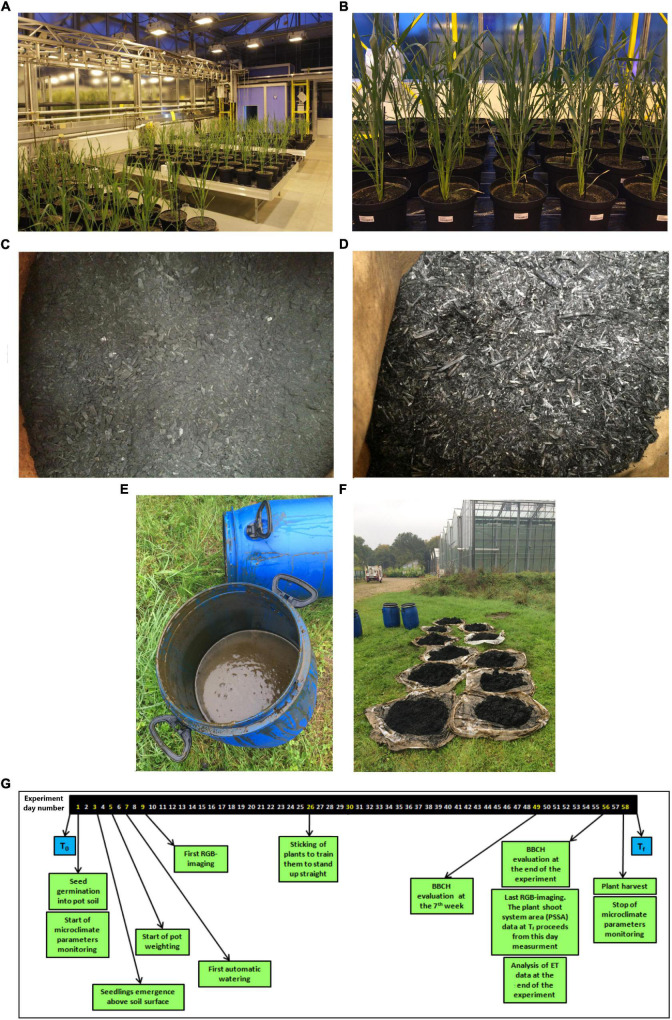
**(A)** The 150 analyzed pot plants, positioned in three flood tray tables with 50 plants each, grown in the ScreenHouse on day 42; **(B)** plants on day 56 close to the end of the experiment, each pot being labeled with a code allowing for complete randomization; appearance of the biochar from **(C)** wood chips (B1) and **(D)** from wheat straw (B2), as provided by the producers; digestate solution contained in a 60-L bin where biochar [specifically for the treatments B1 and B2 incubated with digestate (B1D and B2D, respectively)] was previously dipped, wrapped in a tissue, for 10 days **(E)**; **(F)** drying of the biochars after incubation in the digestate outdoor overnight; **(G)** timeline of the experiment conducted in the ScreenHouse. T_0_, beginning of the experiment; T_f_, end of the experiment; ET, evapotranspiration.

The experiment included five durum wheat genotypes (Duilio, Grecale, Iride, Marco Aurelio, and Saragolla). The following four treatments were performed: B1, with non-activated biochar from wood chips; B1D, with digestate-activated biochar from wood chips; B2, with non-activated biochar from wheat straw; B2D, with digestate-activated biochar from wheat straw. These treatments have been tested in relation to a negative control (C-), corresponding to the soil substrate (SS) lacking any biochar treatment. The control pots (C-) were filled only with 90% SS and 10% silica sand (expressed as dry weight percentages), previously mixed thoroughly in a mechanical mixer. The sample pots for the different biochar treatments were filled with 80% SS, 10% biochar (either pure or previously incubated with digestate), and 10% silica sand. Silica sand has been added to increase drainage. Each sample was replicated 6 times, resulting in 150 potted plants in total, using one plant per pot (Figures 1A,B). All the pots (5 L, 23 cm top diameter, 17 cm base diameter, 18 cm depth) were arranged in a completely randomized factorial design in the ScreenHouse.

Microclimate inside the greenhouse was monitored by sensors for relative humidity (RH, in%), temperature (T, in °C), and photosynthetic active radiation (PAR, expressed as photosynthetic photon flux density, in μmol⋅m/s). Supplemental light was provided to ensure 400 microE/m^2^ at plant level during the day when incoming natural radiation was not sufficient.

### Plant Material

Five Italian durum wheat varieties, commonly cultivated in the Peninsula for their high yield and remarkable commercial impact, were used in this study: Duilio and Marco Aurelio were kindly provided by Società Italiana Sementi (SIS), and Grecale, Iride, and Saragolla were kindly provided by Società Produttori Sementi, which is now Syngenta. Table 1 reports the information provided by the respective seed companies on major qualitative and morpho-physiological traits of interest of these five durum wheat varieties.

**TABLE 1:** Main qualitative and morpho-physiological traits, and yield potential of the five durum wheat varieties used in the phenotyping experiment, as reported by their respective seed companies.

Durum wheat varieties	Qualitative traits					Morpho-physiological traits		Yield
	Hectoliter weight	1000 kernel weight (g)	Protein content (ss%)	Yellow index	Gluten index	Time of spiking	Height	Yield potential
Duilio	Good	47–52	Medium	Medium	Medium	Early	Medium-low	Very good
Marco Aurelio	Good	53–58	Excellent	Optimum	Optimum	Average	Medium	Exceptional
Grecale	Good	>40	High > 14.0	26–28	83%	Early	88 cm	Medium
Iride	High	>44	Medium > 12.0	23–25	83%	Early	85 cm	Optimum
Saragolla	High	>44	Medium > 12.5	25–27	94%	Early	86 cm	Optimum

*Information from www.sisonweb.com and https://www.nxtbook.com/syngenta/Syngenta_Italy/Syngenta_Italia_Catalogo_Generale_2015/index.php?startid=65#/p/1 (last access on September 17, 2021).*

### Soil Substrate, Biochar, and Digestate

The soil substrate (SS) used in all the samples was a commercially available mixture of peat, sand, and pumice (namely SoMi 513, Dachstaudensubstrat; Hawita, Vechta, Germany; Table 2, left).

**TABLE 2:** Main physical-chemical traits of the single separated potting soil components [silica sand, soil substrate (SS), wood chip biochar (B1), B1 incubated with digestate (B1D), wheat straw biochar (B2), and B2 incubated with digestate (B2D)] before mixing (on the left); and of the potting soil mixtures of the control and all treatments at the beginning (T_0_) and at the end (T_f_) of the experiment (respectively, in the center and on the right).

	Potting soil components (before mixing with each other)						Potting soil mixtures at T_0_					Potting soil mixtures at T_f_				
	Silica sand	SS	B1	B1D	B2	B2D	Control	B1	B1D	B2	B2D	Control	B1	B1D	B2	B2D
Dry substance (%)^a^	99.0	65.6	86.7	54.2	67.1	24.6	69.9	69.0	69.2	47.4	66.5	61.0	61.8	60.5	58.1	57.9
Dry bulk density (g/L)^b^	1502	466	366	312	91	122	520	483	493	371	445	523	475	464	381	432
pH in CaCl_2_^3c^	4.9	6.3	8.5	8.2	9.7	8.9	6.0	7.2	6.8	7.6	6.8	6.5	7.3	7.1	7.5	6.9
Electrical conductivity (EC) in H_2_O (μS/cm)^d^	25	587	880	1,301	7,276	3,608	589	475	608	349	841	386	284	325	585	596
KClsalt in H_2_O (g/L)^d^	0.15	2.20	1.94	3.97	5.18	9.47	2.31	1.75	2.28	1.41	2.95	1.71	1.15	1.30	2.03	2.31
KClsalt in CaSO_4_ (g/L)^e^	<0.10	1.28	1.14	2.30	3.36	5.55	1.34	0.98	1.36	0.78	2.34	0.86	0.54	0.69	1.44	1.71
Nitrogen (N) in CAT (mg/L)^f^*	<2	293	<2	31	<2	308	320	151	268	85	413	196	45	105	114	264
Ammonium-nitrogen (NH_4_-N) in CAT (mg/L)^f^	<1	11	<1	30	1	304	8	9	7	10	3	5	6	2	2	2
Nitrate-nitrogen (NO_3_-N) in CAT (mg/L)^f^	<1	282	<1	1	<1	4	312	142	261	75	410	191	39	103	112	262
Phosphorus (P_2_O_5_) in CAT (mg/L)^f^	<2	96	16	79	18	111	77	86	95	243	176	81	55	57	205	158
Potassium (K_2_O) in CAT (mg/L)^f^	<4	378	1,902	3,268	4,655	6,800	322	593	697	1,196	1,524	258	532	562	1,729	1,152
Magnesium (Mg) in CAT (mg/L)^f^	<2	209	71	82	11	58	209	189	200	168	192	203	180	175	155	178

*CAT, Extraction of calcium chloride/DTPA (CAT) soluble elements.*

*^a^VDLUFA Methodenbuch Band I, 1991, A 2.1.1 (Akkr).*

*^b^VDLUFA Methodenbuch Band I, 1991, A 13.2.1 (Akkr).*

*^c^VDLUFA Methodenbuch Band I, 1991, A 5.1.1 (Akkr).*

*^d^VDLUFA Methodenbuch Band I, 1991, A 10.1.1 (Akkr).*

*^e^VDLUFA Methodenbuch Band I, 1991, A 13.4.2 (Akkr).*

*^f^VDLUFA Methodenbuch Band I, A 13.1.1 bzw. A 6.4.1 (Akkr).*

*Here, nitrogen (N) content is the sum of ammonium nitrogen (NH_4_-N) and nitrate nitrogen (NO_3_-N).

Two types of biochar obtained from two very contrasting feedstocks were used for the treatments in this study: B1 from wood chips and B2 from common wheat straw (Figures 1C,D, respectively); both were pyrolyzed in a “Schottdorf”-type reactor, and were kindly provided by Carbon Terra GmbH (Wallerstein, Germany). In particular, B1 was taken from the company’s GMP standard production, providing a certified biochar for animal feed. During its pyrolysis, biomass is first dried and then heated continuously up to 800°C for 36 h in an oxygen-free atmosphere; then, it reaches the oxidation zone, where 15% of the material is burned off and the rest falls through the grid. Thereafter, the resulting biochar is sprayed with water to stop the process, and leaves the system with approximately 20% humidity. Regarding B2, the straw is put into a 2-m^3^ steel box, tightly covered, and heated up to 750°C for 8 h; then it is sprayed with water to stop the process.

B1 is a reproducible type of biochar with homogeneous quality, and its composition and production procedure are well described. Analytical parameters of B1 are reported in Supplementary Table 1A of Kammann et al. (2015).

Here, the main chemical-physical properties of both B1 and B2 were again assessed (Table 2, left). Both types of biochar had alkaline pH (B1 8.5, B2 9.7). According to its woody feedstock, B1 showed > 4 times higher bulk density values, meaning lower porosity, than B2. Their electrical conductivity (EC) in water differed greatly, with that of B2 being > 8 times higher than that of B1 (Table 2, left).

In order to investigate their effects on plants, the two types of biochar were added to the SS either in their pure (B1 and B2 treatments) or previously activated form, i.e., spiking with nutrients, using digestate (B1D and B2D treatments). In practice, for nutrient spiking, a digestate from a commercial biogas facility operating with maize silage was used, as described in previous studies (Robles-Aguilar et al., 2019; Dietrich et al., 2020). Briefly, the fresh digestate used consisted of 7.2% dry matter and 5.3% organic substance. It contained 0.53% N (of which 0.32% was ammonium-N), with a C/N ratio of 6. Furthermore, 0.14% phosphorus, 0.68% potassium, 0.037% magnesium, 0.16% calcium, and 0.03% sulfur were detected. For the activation process, each biochar was wrapped into a stable permeable tissue and submerged in a 60-L bin filled with digestate, allowing the liquid, nutrient-rich fraction to penetrate and soluble nutrients to be absorbed by the biochar (Dietrich et al., 2020; Figure 1E). After 10 days of incubation, each biochar was partially open air dried overnight (Figure 1F). After the treatment with digestate, both B1D and B2D showed lower dry substance content (−37.5 and −63.3% for B1 and B2, respectively), and low pH reduction (<8.3%) than the pure biochar (Table 2, left). The digestate treatment had a minor opposite effect on dry bulk density, which showed slighter reduction (−14.8%) in B1D than in B1, and increase (+34.1%) in B2D compared with B2 (Table 2, left). N content (mainly present in the ammonium form) increased considerably in both nutrient-spiked types of biochar, from less than 2 mg/L in both types of biochar up to 31 mg/L in B1D and 308 mg/L in B2D. In a similar way, the content of main macronutrients also increased notably. In particular, P was > 79.8%, and K was > 31.5% in both nutrient-spiked types of biochar. Concerning Mg, its increase was very high (81%) in B2D and more moderate (13.4%) in B1D (Table 2, left).

To supply the pots with precise amounts of each component (SS, silica sand, B1, B1D, B2, and B2D), their moisture content was measured with HB43-S Moisture Analyzer (Mettler Toledo, Gießen, Germany). Soil analyses of the potting components before mixing with each other (Table 2, left) and of the potting mixtures of the control and each treatment (B1, B1D, B2, and B2D) at the beginning of the experiment (before seed germination, T_0_) as well as the end (T_f_) after 58 days (Table 2, center and on the right, respectively), were performed according to the procedures applied by LUFA NRW, Landwirtschaftskammer Nordrhein-Westfalen (VDLUFA, 1991).

### Plant Growth and Phenotyping

Three seeds of uniform size and weight per pot were put to germinate directly in the soil substrate, suitably spaced from each other 3 cm below the air-soil interface. After 3 days, only one plantlet per pot was kept, and the other two were removed. The pots were irrigated three times per week to keep soil moisture level around a 50% water holding capacity throughout the experiment with an automated watering system. Pot water loss due to plant evapotranspiration (ET) was recorded by weighing the pots before watering, ensuring equal soil moisture levels. Given the relatively high percentage of humidity inside the ScreenHouse, we assumed that most of the weighed water loss was related to plant transpiration.

The imaging station in the ScreenHouse allowed capturing data on leaf area expansion, inferred from the number of green pixels in the image belonging to the plant (Golzarian et al., 2011). The phenotyping system allowed repeated measures over the projected shoot system area (PSSA). In correspondence with the ET measures, each plant was imaged (RGB) for dynamic estimation of shoot biomass (as projected shoot system area, PSSA) three times per week. After each measurement, the pots were automatically re-randomized with a laser positioning system and a robotic crane to avoid any systematic bias from position within the greenhouse. The subsequent imaging processing pipeline has been described earlier by Nakhforoosh et al. (2016). Plant growth was evaluated in the early vegetative stage during the course of the experiment for a total of 58 days until the experiment was stopped (Figure 1G).

Additional plant traits were measured to aid plant behavior evaluation (Table 3). Plant phenological developmental stage was observed throughout the experiment and evaluated by BBCH-scale (Meier, 1997), acronym for Biologische Bundesanstalt für Land- und Forstwirtschaft, Bundessortenamt und CHemische Industrie, 7 weeks after seed germination and at the end of the experiment. At the end of the experiment (T_f_), the number of tillers per plant (tiller number, TN), number of spikes per plant (spike number, SN), spike length (SL), and plant height (PH) from the base of the stem up to the end of the emerging spike were measured. After that, aboveground tissue was excised, and shoot fresh weight (FW) was annotated. Plant shoot area (PSA_Licor_) was also measured with a LI-3100C Area Meter (LI-COR, Inc., Bad Homburg, Germany), and then shoot dry weight (DW) was finally recorded after 48 h of drying at 75°C. Actual water content (WC) of the aboveground plant body was calculated by the formula WC = 100*(FW-DW)/FW.

**TABLE 3:** List of plant phenotypic traits measured.

Abbreviation	Description	Type of plant trait	Unit of measure	Measurement period
PSSA	Projected Shoot System Area	Morphological	cm^2^	Three times/week throughout the experiment
PSA_Licor_	Total plant shoot area by Licor area meter	Morphological	cm^2^	T_f_
ET	Daily evapotranspiration (as amount of water lost)	Physiological	ml/day	Three times/week throughout the experiment
BBCH	BBCH-plant phenology scale	Phenological	–	T_7 weeks_, T_f_
TN	Number of tillers	Agro-morphological	n°	T_f_
SN	Number of spikes	Agro-morphological	–	T_f_
SL	Spike length	Agro-morphological	cm	T_f_
PH	Plant height	Agro-morphological	cm	T_f_
FW	Plant aboveground fresh weight	Agro-physiological	g	T_f_
DW	Plant aboveground dry weight	Agro-physiological	g	T_f_
WC	Plant aboveground water content	Physiological	%	T_f_

### Statistical Analyses

All the statistical analyses were conducted with the IBM SPSS Statistics 23 software. Data were analyzed for their normality and equality of variances by the Shapiro-Wilk test and the Levene test, respectively. When these two conditions were assessed, a two-way ANOVA was carried out for the dependent variables, represented by the plant-related traits, with “genotype” and “treatment” as fixed factors (independent variables). The Ryan-Einot-Gabriel-Welsch-and-Quiot (REGWQ) method with Bonferroni correction was used for *post hoc* testing. Differently, when a dataset did not meet the criteria of the equality of variances, a one-way ANOVA was performed with Welch correction and the Games-Howell method for *post hoc* testing. The selected statistical significance, depending on the data and test, is reported case by case.

A bivariate Pearson correlation analysis was conducted for the plant trait datasets, after assessing that they did not violate the assumptions of data normality and homoscedasticity, with a two-tailed test and *p* < 0.01. In a different way, the correlation between the two time-series datasets of plant ET and PSSA was examined with the non-parametric Spearman coefficient.

## Results

### Potting Soil Analysis

When observing the potting soil mixtures tested (SS, i.e., C-, B1, B1D, B2, and B2D), and comparing their main parameters at the beginning (T_0_) and the end of the experiment (T_f_) (Table 2, middle and on the right, respectively), C- showed the highest dry bulk density values among all the biochar treatments, which means it has low porosity. As a general behavior, in the analyzed samples, while dry bulk density remained almost constant, the dry substance tended to decrease during the experiment. A relevant exception to this trend was represented by the B2 mixture, which showed the lowest values of dry substance content (47.4% at T_0_ that differently from the general trend increased up to 58.1% at T_f_) and dry bulk density (371 g/L at T_0_ that did not vary considerably at T_f_, as for the other treatments).

As usual, the addition of biochar in the soil substrate resulted in pH increase more pronounced in the mixture with pure biochars than in those with digestate-treated biochars. There were no marked pH changes from the beginning to the end of the experiment, and all the SSs and their mixtures used could be classified as neutral or weak alkaline, with values ranging from 6 to 6.5 in C- and up to 7.5 to 7.6 in B2 (Table 2). Electrical conductivity (EC) in water, and KCl salt in water as well as in calcium sulfate showed a general decrease at the end of the experiment, but for these traits there was again the exception of the B2 mixture, whose starting values were always lower than in the other soil mixtures, and different from the general fashion they increased considerably at the end (in particular, EC in water of B2 was 40.8% lower than C- at T_0_ and 51.6% higher than C- at T_f_, increasing 1.7 times during the experiment duration). It is also noticeable that the highest values of these three traits were detected in the mixture B2D, both at T_0_ and at T_f_ (in particular, EC in water of B2D was 42.8% higher than C- at T_0_ and 35.2% higher than C- at T_f_, decreasing 1.4 times during the experiment duration), respectively, 841 and 596 μS/cm for EC, 2.95 and 2.31 g/L for KCl salt in H_2_O, and 2.34 and 1.71 for KCl salt in CaSO_4_; Table 2).

The main nutrients (N, P, K, and Mg) showed obvious reduction at the end of the experiment with respect to T_0_, with few exceptions. Unexpectedly, in B2, an increase in nitrate-nitrogen and in potassium (K) was detected from T_0_ to T_f_. It is also noticeable that the soil mixtures with wheat straw biochar, either as pure (B2) or incubated in the digestate (B2D), resulted in higher concentration of phosphorous (from 2 up to 3.2 times more than in C-) and K (from 3.7 up to 6.7 times more than in C-) (Table 2).

### Plant Growth Performance

#### Plant Phenology

The seeds showed a high percentage of germination (> 95%). Concerning the developmental stage, almost at the end of the experiment (day 56), terminated on day 58 (Figure 1G), all the plants were about to complete the heading stage (BBCH 5) or, in many cases, the beginning of the flowering stage (BBCH 6). The analysis of BBCH data on day 49 (7 weeks after seed germination) and on day 56 provided comparable results. One-way ANOVA showed that there was a significant (*p* < 0.001) effect of both “genotype” and “treatment.” The analysis using “genotype” as fixed factor showed the highest BBCH values for Grecale and Marco Aurelio, the lowest for Iride, Duilio and Saragolla in between. On the other hand, the analysis using “treatment” as fixed factor showed the highest BBCH values for B2 and B2D. C-, B1 and B1D showed lower values compared with B2 and B2D, but similar values among each other (Supplementary Material 1).

#### Plant Aboveground Surface Area

**FIGURE 2:**
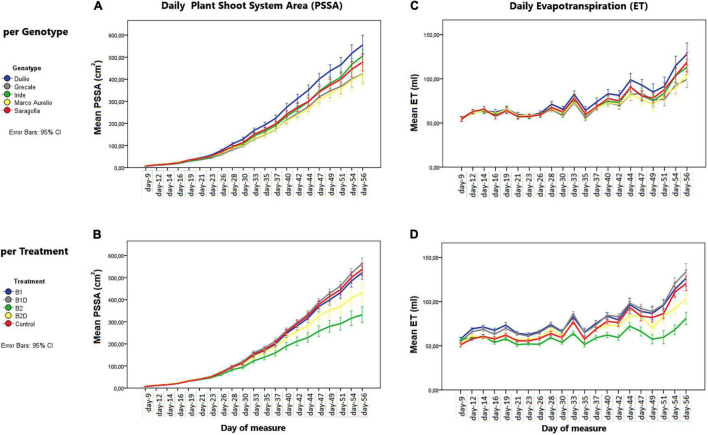
**(A,B)** Plant shoot system area (PSSA) per day (in cm^2^) on the left and **(C,D)** evapotranspiration (ET) by the “pot + plant” system per day (in ml) on the right. The dynamic trends are displayed by these two parameters, per day of measure, in the **(A,C)** different genotypes and **(B,D)** different treatments. Bars represent mean values, with error bars denoting 95% confidence interval (CI).

**FIGURE 3:**
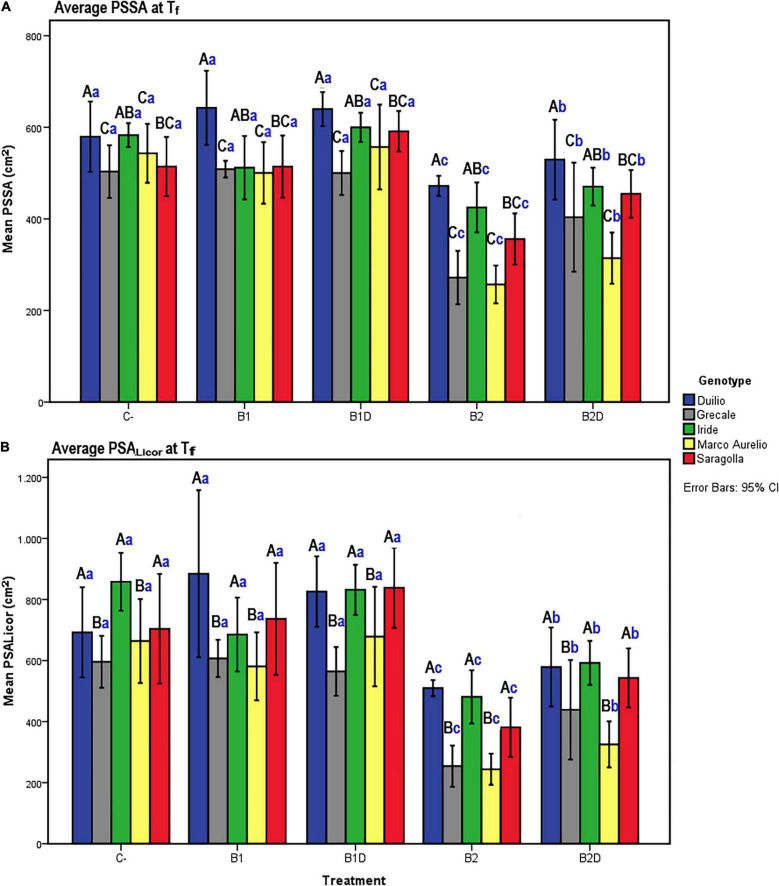
**(A)** PSSA and **(B)** plant shoot area measured with Licor area meter (PSA_Licor_) at T_f_ as a function of the genotype and treatment. Bars represent mean values, with error bars denoting 95% confidence interval (CI). Different bold letters indicate significant difference according to the R-E-G-W-Q test at the *p* < 0.001 level, with the black uppercase letters referring to the “genotype” subset and the blue lowercase ones to the “treatment” subset (Supplementary Material 2).

From the analysis of the daily mean values corresponding to the day of measure, the PSSA showed a linear (positive) growth dynamic trend, influenced by “genotype” as well as “treatment” (Figures 2A,B, respectively). Starting from approximately 30 days after sowing, significant differences in PSSA were observed among the genotypes and treatments. These differences persisted and became more significant and evident at the end of the experiment, with Duilio reaching the highest average value of 573 cm^2^ and Marco Aurelio the lowest one of 434 cm^2^ with respect to the other genotypes, and with B1D reaching the highest average value of 578 cm^2^ and B2 the lowest one of 356 cm^2^ with respect to the other treatments (see below the analysis on T_f_; Figure 3A and Supplementary Material 2).

To assess that the RGB imaged shoot biomass data accurately reflected the real plant aboveground area, a bivariate correlation analysis between the PSSA at T_f_ (PSSA on day 56) and the plant shoot area measured at T_f_ with the Licor area meter after destructive harvest of the plants (PSA_Licor_) was performed. The Pearson coefficient was very high (*r* = 0.94, *p* < 0.01), indicating a very strong positive correlation between the two variables at T_f_ (Table 4), even in the presence of general slight underestimation of PSSA compared to PSA_Licor_.

**TABLE 4:** Results of the bivariate Pearson correlation analysis of the plant phenotypic traits measured at the end of the experiment (T_f_): projected shoot system area (PSSA), total plant shoot area with Licor area meter (PSA_Licor_), evapotranspiration (ET), plant height (PH), plant aboveground fresh weight (FW), dry weight (DW), and water content (WC).

Plant trait	PSSA	PSA_Licor_	ET	PH	FW	DW	WC
PSSA	–						
PSA_Licor_	0.941**	–					
ET	0.792**	0.795**	–				
PH	0.305**	0.209**	0.297*	–			
FW	0.957**	0.979**	0.803**	0.197*	–		
DW	0.928**	0.851**	0.736**	0.263**	0.919**	–	
WC	0.740**	0.841**	0.628**	0.020	0.799**	0.538**	–

*N = 150 (except for PH, N = 148).*

**Correlation is significant at the 0.01 level (two-tailed).

*Correlation is significant at the 0.05 level (two-tailed).

At the end of the experiment, the two-way ANOVA conducted for both PSSA and PSA_Licor_ showed that there was a highly significant (*p* < 0.001) main effect of both “genotype” and “treatment” on plant aboveground surface area, and that the interaction between “genotype” and “treatment” was only statistically significant (*p* < 0.01) for PSSA but not significant (*p* > 0.05) for PSA_Licor_ (Table 5). When using “genotype” as fixed factor, PSSA was significantly higher in Duilio than all the other genotypes, followed by Iride and Saragolla (not significantly different between each other), and then by Grecale and Marco Aurelio (not significantly different between each other). When using “treatment” as fixed factor, B2 was related to the lowest PSSA (<34.6% than C-), followed by B2D (significantly different from B2 and all the other treatments, PSSA < 20.2% than C-). B1 did not show any significant effect on PSSA either as nutrient-spiked, and indeed B1 and B1D clustered together with C- (Figure 3A). Comparing PSSA with PSA_Licor_, they differed slightly only with respect to the “genotype” factor, where PSA_Licor_ data of Duilio clustered together with Iride and Saragolla (Figure 3B). The descriptive statistics results are schematically shown by two histograms, one for PSSA and one for PSA_Licor_ (Figures 3A,B, respectively; see also Supplementary Material 2 for technical details).

**TABLE 5:** Results of the two-way analysis of variance (ANOVA) (tests of between-subjects effects) on PSSA, PSA_Licor_, and ET data at the end of T_f_, with “genotype” and “treatment” as fixed factors.

Dependent variable	Source	Sum of squares	df	Mean square	F	Sig.
PSSA	Corrected model	1.562E12	24	6.508E10	18.798	0.000
	Genotype	4.045E11	4	1.011E11	29.207	0.000
	Treatment	1.010E12	4	2.525E11	72.940	0.000
	Genotype*Treatment	1.473E11	16	9,207,862,582	2.660	0.001
	Error	4.328E11	125	3,462,162,359		
	Corrected total	1.995E12	149			
PSA_Licor_	Corrected model	4725724.630	24	196905.193	13.997	0.000
	Genotype	1236858.011	4	309214.503	21.980	0.000
	Treatment	3122322.165	4	780580.541	55.486	0.000
	Genotype*Treatment	366544.455	16	22909.028	1.628	0.071
	Error	1758517.947	125	14068.144		
	Corrected total	6484242.577	149			
ET	Corrected model	77275.208	24	3219.800	9.180	0.000
	Genotype	15152.842	4	3788.210	10.801	0.000
	Treatment	54063.149	4	13515.787	38.535	0.000
	Genotype*Treatment	8165.387	16	510.337	1.455	0.128
	Error	43492.000	124	350.742		
	Corrected total	120767.208	148			

*df, degree of freedom; F, F-statistic (F = variation between sample means/variation within the samples); Sig., p-value.*

### Plant Evapotranspiration

**FIGURE 4:**
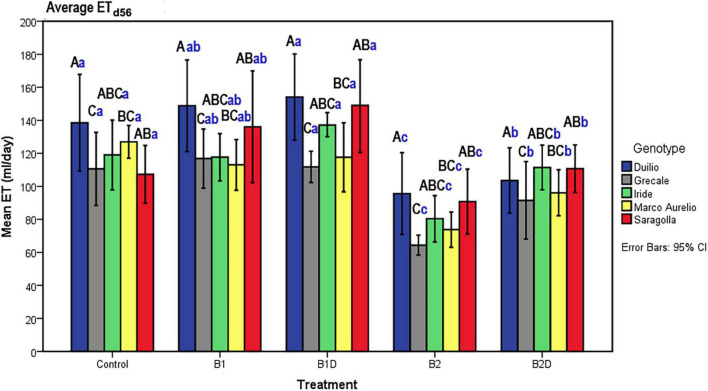
ET on day 56, close to T_f_, as a function of genotype and treatment. Bars represent mean values, with error bars denoting 95% CI. Different bold letters indicate significant differences according to the R-E-G-W-Q test at the *p* < 0.001 level, with the black uppercase letters referring to the “genotype” subset and the blue lowercase ones to the “treatment” subset (Supplementary Material 4).

Plant evapotranspiration (ET) was estimated as ml of water loss by weighting the pots throughout the course of the experiment; As observed for the PSSA plant trait, ET also showed an overall increasing dynamic trend, but with relevant fluctuations shaping a zigzag distribution (Figures 2C,D), possibly related to the inter-day smooth variations under the microclimate conditions (T, RH, and PAR) inside the ScreenHouse (Supplementary Material 3). At the beginning of the experiment, the daily ET from each pot was around 50 ml in all the samples, and then it increased, as expected, with the growth of the plants and extension of plant surfaces available for the transpiration process. The ET dataset on day 56, close to the end of the experiment, was analyzed by two-way ANOVA, evidencing statistically significant differences due to “genotype” as well “treatment” (*p* <0.001), but not due to the “genotype*treatment” interaction (Table 5 and Supplementary Material 4). Focusing on “genotype,” ET_d56_ in Duilio was significantly higher (*p* <0.001) than in Grecale and Marco Aurelio (>22.5 and >17.7%, respectively) and in Iride and Saragolla, which even had lower significance (*p* <0.05). Focusing on “treatment,” B2 negatively affected ET (36% lower than the C-), and indeed it was significantly different from the control and all the other samples (*p* <0.001); ET in B2D was significantly lower than that in B1 and B1D (*p* <0.001), and had lower probability than C- (*p* <0.05) (Figure 4).

### Agro-Morphological and Agro-Physiological Plant Traits

The plant traits related to wheat morphology and yield-tiller number (TN), spike number (SN), spike length (SL), and plant height (PH) were measured at T_f_ to aid in plant behavior evaluation (Table 3). The results of the one-way ANOVA showed that no significant differences were observed in SN and in SL, and that TN showed differences among the genotypes and treatments. In particular, Marco Aurelio, among the genotypes, and B2, among the treatments, showed the lowest average tiller number (*p* >0.05, data not shown).

**FIGURE 5:**
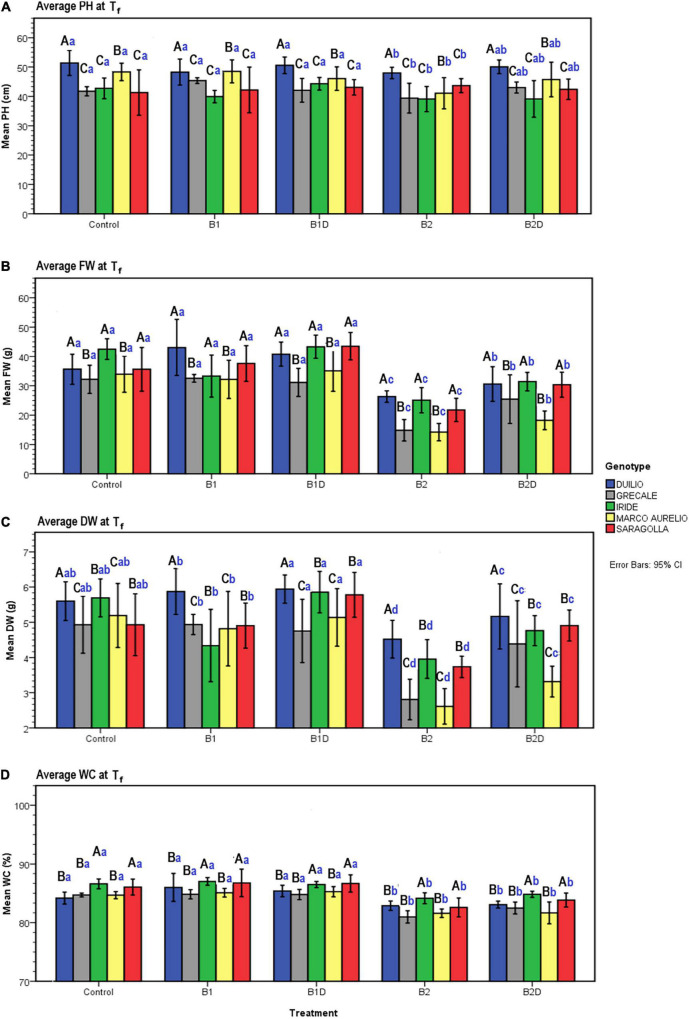
**(A)** Plant height (PH), **(B)** plant aboveground fresh weight (FW), **(C)** dry weight (DW), and **(D)** water content (WC) measured data at T_f_ as a function of genotype and treatment. Bars represent mean values, with error bars denoting 95% confidence intervals (CI). Different bold letters indicate significant difference according to the Ryan-Einot-Gabriel-Welsch-and-Quiot (R-E-G-W-Q) test at the *p* < 0.05 level for PH and DW and at the *p* < 0.01 level for FW and WC, with the black uppercase letters referring to the “genotype” subset and the blue lowercase ones to the “treatment” subset.

Concerning PH, resulting from the two-way ANOVA, a highly significant effect of “genotype” (*p* <0.001) and a slightly significant effect of “treatment” (*p* <0.05) was observed, while the interaction term “genotype*treatment” was not statistically significant (*p* >0.05) (Table 6). The *post hoc* results of PH data analysis are reported in the form of histogram in Figure 5A. With respect to the “genotype” factor, PH in Duilio was significantly higher than that in all the other genotypes, followed by Marco Aurelio; then, Grecale, Iride and Saragolla presented the lowest values, similar among each other. With respect to the “treatment” factor, B1 and B1D did not affect PH. Moreover, while B2 had a negative effect on PH, when incubated with digestate (i.e., B2D) the PH reduction was less pronounced (Figure 5A).

**TABLE 6:** Results of the two-way ANOVA (tests of between-subjects effects) on PH, and plant aboveground FW, DW, and WC data at T_f_, with “genotype” and “treatment” as fixed factors.

Dependent variable	Source	Sum of squares	df	Mean square	F	Sig.
PH	Corrected model	1963.068	24	81.794	5.317	0.000
	Genotype	1431.343	4	357.836	23.263	0.000
	Treatment	172.310	4	43.078	2.800	0.029
	Genotype*Treatment	348.712	16	21.795	1.417	0.144
	Error	1892.012	123	15.382		
	Corrected total	3855.080	147			
FW	Corrected model	10037.546	24	418.231	16.246	0.000
	Genotype	2204.992	4	551.248	21.413	0.000
	Treatment	6935.728	4	1733.932	67.355	0.000
	Genotype*Treatment	896.826	16	56.052	2.177	0.009
	Error	3217.900	125	25.743		
	Corrected total	13255.446	149			
DW	Corrected model	118.023	24	4.918	10.842	0.000
	Genotype	27.762	4	6.940	15.302	0.000
	Treatment	72.858	4	18.215	40.158	0.000
	Genotype*Treatment	17.403	16	1.088	2.398	0.004
	Error	56.697	125	0.454		
	Corrected total	174.719	149			
WC	Corrected model	438.170	24	18.257	14.838	0.000
	Genotype	114.798	4	28.699	23.325	0.000
	Treatment	302.675	4	75.699	61.498	0.000
	Genotype*Treatment	20.697	16	1.294	1.051	0.409
	Error	153.804	125	1.230		
	Corrected total	591.974	149			

*df, degree of freedom; F, F-statistic (F = variation between sample means/variation within the samples); Sig., p-value.*

Additionally, the agro-physiological traits related to the aboveground plant tissues, fresh weight (FW), and dry weight (DW), besides water content (WC), were evaluated at T_f_. The results of the two-way ANOVA are reported in Table 6. FW showed a highly significant effect of both “genotype” and “treatment” as well as the “genotype*treatment” interaction (*p* < 0.01); and the same was observed for DW, even though the interaction was more significant (*p* < 0.005). WC showed a significant effect of both “genotype” and “treatment,” but not of the interaction term. The resulting histograms from mean values and related errors of these traits are also represented in Figure 5. With respect to “genotype,” Duilio, Iride, and Saragolla showed a higher FW (35.3, 35.1, and 33.8 g, respectively) than Marco Aurelio and Grecale (27.2 and 26.7 g, respectively). With respect to “treatment,” B2 had a reliable 43.2% reduction in FW compared C-, which was slightly relieved (24.3%) by the digestate treatment in B2D, while the application of woody biochar (B1 and B1D) did not significantly affect FW with respect to C- (Figure 5B). In general, the DW results reflected those of FW, as expected, with Duilio (5.42 g) exhibiting the highest DW values, followed by Iride and Saragolla (4.92 and 4.85 g, respectively), and then by Grecale and Marco Aurelio (4.36 and 4.21 g, respectively) with the lowest DW values. At the same time, the DW results distributed similarly to FW also in relation to the treatments, with B2 being related to the lowest DW values (66.9% lower than C-), followed by B2D (14.5% lower than C-; Figure 5C).

Regarding WC, even though only low-entity significant differences were observed, Grecale and Marco Aurelio showed the lowest WC percentages together with Duilio (83.6, 83.7, and 84.3%, respectively), which for the first time appeared to cluster with these genotypes, while Saragolla and Iride presented a higher WC (85.2 and 85.8%, respectively). Concerning the treatments, B2 and B2D resulted in an inferior plant tissue hydration level (3.2 and 2.4% lower than C-, respectively; Figure 5D).

### Correlations Among the Evaluated Plant Phenotypic Traits

Bivariate Pearson correlation analysis was performed for the following plant variables at the end of the experiment: PSSA, PSA_Licor_, ET, PH, FW, DW, and WC; the resulting correlation matrix is reported in Table 4. Besides PSSA with PSA_Licor_, strong positive linear relationship correlations (*p* > 0.01) were ascertained among most of the analyzed plant phenotypic traits. The only exceptions were represented by FW with PH showing a positive correlation with lower significance (*p* > 0.05), and by WC with PH, whose result did not correlate at all. As it can be expected, ET resulted to be directly proportional to all the plant traits, except for PH that anyhow demonstrated no link with the other traits. Among the variables analyzed here, the lower moderate correlation was that between DW and WC.

A schematic representation summarizing the effects of both “genotype” and “treatment” on the analyzed plant phenotypic traits is shown in Table 7. The “genotype” effect is shown (Table 7, left) using Duilio as the reference, while the control was used as reference for summing up the “treatment” effect (Table 7, right). Accordingly, while Iride and Saragolla behaved like Duilio with respect to PSSA, PSA_Licor_, ET, FW, and DW, always showing higher values for these traits, in Grecale and Marco Aurelio, these traits were negatively influenced. Duilio, in particular, often showed the highest values, for DW it even clustered apart with values significantly higher than those for Iride and Saragolla (*p* < 0.01). On the other hand, the wood biochar, both as pure and after nutrient spiking with digestate (B1 and B1D, respectively), behaved like the control in promoting plant growth, while the wheat straw biochar, both in B2- and in B2D-treated plants, negatively affected plant growth.

**TABLE 7:** Schematic representation of the genotype effect on the analyzed plant phenotypic traits at the end of the experiment (T_f_) in terms of increase or decrease of the trait value with respect to Duilio, on the left side, and of the biochar treatment effect with respect to the control, on the right side.

Plant traits (at T_f_)	“Genotype” effect with respect to Duilio				“Treatment” effect with respect to the control			
	Grecale	Iride	Marco Aurelio	Saragolla	B1	B1D	B2	B2D
BBCH	↑	↓	↑	—	—	—	↑	↑
PSSA/PSA_Licor_	↓	—	↓	—	—	—	↓	↓
ET	↓	—	↓	—	—	—	↓	↓
PH	↓	↓	↓	↓	—	—	↓	—
FW	↓	—	↓	—	—	—	↓	↓
DW	↓	—	↓	—	—	—	↓	↓
WC	—	↑	—	↑	—	—	↓	↓

*BBCH, BBCH plant phenology scale; PSSA, projected shoot system area; PSA_Licor_, plant shoot area measured by Licor area meter; ET, daily evapotranspiration; PH, plant height; FW, plant aboveground fresh weight; DW, dry weight; WC, water content. B1, Biochar from wood chips; B1D, B1 incubated with digestate; B2, biochar from wheat straw; B2D, B2 incubated with digestate.*

*“↑” indicates significant augmentation (of any intensity) with respect to Duilio.*

*“↓” indicates significant reduction (of any intensity) with respect to Duilio.*

*“—” indicates no significant variation with respect to Duilio.*

A positive correlation was also found in the measurement of ET and PSSA repeated over time (Figure 2), probably not related in a linear fashion, and resulting in a Spearman’s rho equal to 0.679 (*p* < 0.01, two-tailed; Supplementary Material 5).

## Discussion

Soil amendment with biochar is considered a good agricultural practice (Laird, 2008). Many research studies encompassing greenhouse and field trials have already been committed to this topic over the last 20 years (Shaaban et al., 2018; Purakayastha et al., 2019).

The main aim of this study was to evaluate the effects due to difference in Italian commercial varieties of durum wheat (genotype effect) and those due to different biochar amendments (treatment effect) on plant growth performance. To do so, we employed state-of-the-art plant phenotyping devices to gain insights into plant environmental interactions and their translation into applications in crop management practices (Fiorani and Schurr, 2013; Pieruschka and Schurr, 2019). The experiment was arranged in order to broaden awareness of the following aspects: (i) the effect of genotype on the aboveground plant growth-associated traits in the different pot biochar treatments; (ii) the effect of the biochar feedstock material on biochar nutrient content, potting soil mixtures, and resulting plant performance; (iii) the short-time effect of applied pure biochars vs. nutrient-enriched biochars using digestate on plant growth. The obtained results are discussed in relation to the soil physical-chemical properties.

(i) The effect of biochar on plant growth performance is genotype-dependent.

To date only few scientific publications have reported data from comparative analysis conducted on multiple plant genotypes treated with biochar. Most of the researchers who tested at least two different genotypes of a plant species to evaluate the effects of biochar on some specific plant traits shared the result that plant genetic composition is a valuable characteristic that has to be considered when evaluating the potential for crop response to biochar or any other biological soil amendment (Racioppi et al., 2019; Win et al., 2019; Liu et al., 2021). In our study we assessed a genotype-specific effect on plant growth under different tested biochar treatments. Indeed, the five durum wheat genotypes used could be clustered into two groups according to their influence on the different plant shoot traits measured at the end of the experiment, i.e., plant surface area (both PSSA and PSA_Licor_), fresh weight (FW) and dry weight (DW), and water loss by evapotranspiration (ET). The group of Duilio, Iride, and Saragolla always showed higher values for these traits than Grecale and Marco Aurelio (Table 7, left), suggesting a positive influence. It is interesting to underscore that Grecale and Marco Aurelio, which had faster early development, as assessed by their significantly higher BBCH, corresponded to the genotypes in which the aboveground plant traits were negatively affected. On the contrary, Duilio, Saragolla, and Iride, which had been less advanced in development, showed higher values of plant surface area, FW, DW, and ET. Such effect could be foreseen, since plants that complete their development fast are supposed to produce less biomass. Accelerated phenology and the resultant shortening of growth duration can reduce plant performance in terms of produced biomass and yield (Horie et al., 1992).

In a previous study, Latini et al. (2019) has reported that selection of the best favorable combination of biochar type and crop cultivar to be cultivated in a specific soil environment could foster superior yields. They assessed this hypothesis after investigating the impact of wood biochar and wheat straw biochar on plant performance and on rhizosphere microbiota in Italian durum wheat varieties of Duilio and Marco Aurelio: the analysis showed that the combination of straw-based biochar with the Marco Aurelio variety exhibited better growth performance. Unexpectedly, this result is not in agreement with our current finding, since the growth performance of Marco Aurelio was found to be lower than that of the other genotypes, such as Duilio, and particularly in soil mixtures containing wheat straw biochar. This was likely due to the different applied experimental conditions, which included completely different types of potting substrate, very dissimilar properties of the biochars used in the two different studies and, consequently, very diverse nutrient availabilities for the potted plants. In particular, the wheat straw biochar applied in this study had a much higher EC. These different results highlight the complexity of biochar-plant interactions and strengthen our awareness of a variation within-species of the response to biochar amendment, which opens the door to the potential for breeding for a positive biochar response, as suggested by French and Iyer-Pascuzzi (2018).

(ii) The biochar feedstock is broadly responsible for biochar nutrient content and consequent effect on plant growth.

It is already known that biochar properties depend strictly on feedstock sources, production temperature, and residence time and pressure (Zhao et al., 2013; Hassan et al., 2020; Yaashikaa et al., 2020). Here, we selected two biochars produced at a high pyrolysis temperature from distinct feedstocks to be used in the experiment (B1 from wood chips and B2 from wheat straw), to point up the strong influence played by the two feedstocks on plant response to biochar application, which in turn is strictly linked to the final biochar main physical-chemical characteristics in the potting substrate. The results of our analysis focusing on the treatment showed that biochar from wood chips did not show any significant effect on plant growth performance with respect to the control without biochar. Differently, the biochar from wheat straw had a significant negative influence on plant aboveground area (both PSSA and PSA_Licor_), evapotranspiration (ET), fresh (FW), and dry weight (DW) (Table 7, right).

As expected, the biochar-treated soil samples presented augmented porosity with respect to C- (Ippolito et al., 2015), with B2 exhibiting the highest porosity among all the samples throughout the experiment. All the parameters considered in Table 2 varied dramatically between the pure biochar samples B1 and B2. In accordance with several published manuscripts, wood-based biochars as B1 contain more C and lower available plant nutrients than grass-based biochars as B2 (Ippolito et al., 2015; Alkharabsheh et al., 2021). The much higher EC, indicating a higher concentration of dissolved ions and salts, exhibited by the digestate-treated biochar samples (B1D and B2D), both at T_0_ and T_f_, is also an indication of a greater amount of available mineral elements with respect to the treatments with pure biochar (B1 and B2). It is not uncommon to find that high EC has detrimental effects on plants, affecting their equilibrate growth particularly in the early growth stage (Lam et al., 2020; Celletti et al., 2021).

The main soluble nutrient elements decreased at the end of the experiment with respect to T0, presumably due to plant uptake. In the potting soil mixtures at T_0_, the up to 3.76 times decrease of N in B2 compared to the control was probably due to its high surface area and porosity, which absorbed dramatically N from the substrate once added in the solution. Differently, the more than 20% augmentation, compared to the control of N, in the B2D mixture was an effect of the incubation with digestate. It is particularly interesting that before mixing the potting soil components, even if incubated with the same digestate under identical conditions, B1D seemed not to be able to incorporate a similar relevant amount of nitrogen as B2D, whose N concentration resulted about 10 times lower than in B2D. We can hypothesize that the incapability of B1D depends on its reduced cation exchange capacity, as typical in wood biochars with respect to the ones obtained from other feedstocks as the wheat straw (Tomczyk et al., 2020; Alkharabsheh et al., 2021). Furthermore, at T_0_, the samples amended with B2 and B2D contained about double soluble P and K compared with B1 and B1D (Table 2). In our opinion, the negative effects on durum wheat plant growth determined by the treatments B2 and B2D (even though the digestate incubation has been found to determine a small improvement in PSSA and PSA_Licor_, ET, FW, and DW with respect to the application of pure wheat straw biochar) could be traced back to an excessive amount of soil nutrients, like P and K, also considering that the use of the SoMi soil substrate conferred in general a high pre-fertilization level in all the samples. Several studies reported that extra-fertilization was harmful to plants (Li et al., 2019).

(iii) Biochar nutrient-loading by digestate incubation affects plant growth performance depending on biochar nutrient content.

The biochar from plant biomass itself does not contain nutrients, but it provides a permanent soil structure, a pleasant habitat for microorganisms (Okareh and Gbadebo, 2020), and it also assists in fertilizer action (Ding et al., 2016). On the other hand, anaerobic digestate (AD) is typically rich in essential nutrients like nitrogen, especially as NH_4_ (Table 2), and phosphorus, potassium, and magnesium, besides trace elements and organic matter (Tambone et al., 2010; Celletti et al., 2021). Thus, it may replace inorganic fertilizers and maintain grassland productivity with a lower negative environmental impact (Walsh et al., 2012). The increasing number of biogas facilities in the last decade has resulted in vast amounts of digestates, with maize silage being one of the main substrates used across Europe (Robles-Aguilar et al., 2019). In this experiment, we infused the two biochars used in maize silage digestate for 10 days (samples B1D and B2D), with the purpose of increasing their nutrient content and improving plant growth performance and yield, as also reported for other crop species. For example, the application of liquid digestate plus biochar in a tomato-cultivated field led to higher yield than the application of biochar alone or (liquid or pelleted) digestate alone (Ronga et al., 2020). As verified by the analysis, the resulting biochars were strongly nutrient-enriched (Table 2). Anyway, looking at the plant growth-related traits at the end of the experiment, the incubation of digestate showed different effects depending on the type of biochar (Table 7). Such effects shown by the same digestate used for biochar nutrient spiking should be searched not only in the different biochar feedstock, but also in the nutrient content of the amended soil, and the relationships between biochar dosage and the plant growth requirement (Gale and Thomas, 2019).

## Conclusion

In this study, we used an integrated approach combining phenotyping and biochar amendment analysis to follow plant growth dynamics, and to assess possible plant performance improvement. In particular, we evaluated some agro-morphological and agro-physiological traits related to the aboveground plant. We found that plant area, substrate evapotranspiration (ET), fresh weight (FW), and dry weight (DW) were strongly correlated with each other, and that these plant traits disclosed significant genotype dependence, allowing for the clustering of the genotypes in two different groups: (1) with increased values of the abovementioned plant traits, thus exhibiting improved growth performance, like Duilio, Iride, and Saragolla, and (2) with decreased values of such plant traits, like Marco Aurelio and Grecale. Furthermore, concerning the soil-applied treatments, no significant differences were found in the monitored traits between the samples treated with woody biochar and the control ones without biochar. Differently, the wheat straw biochar used in this experiment, characterized by high nutrient content and EC, decreased plant growth performance, evaluated based on plant shoot system area, ET, FW, and DW.

Our findings support the indication that biochar nutrient spiking by incubation with digestate could be considered as a sustainable agricultural practice. Indeed, the biochar from wheat straw incubated with digestate produced a certain relief from the negative effect that the pure biochar (B2) had on the measured plant growth traits, particularly evident when looking at DW, even though the same was not observed for biochar from wood chips incubated with digestate. This is probably due to the lack of any effect of the pure biochar (B1) on these plant traits. Thus, in order to attain an improvement in crop growth performance, it should be performed addressing carefully the crop genotype, feedstock, and physical-chemical properties of both biochar and soil.

## Data Availability Statement

The original contributions presented in the study are included in the article/Supplementary Material, further inquiries can be directed to the corresponding author/s.

## Author Contributions

AL, FF, and NDJ conceived the study and planned the experimental greenhouse setup. AL conducted the experiments and wrote the draft of the manuscript. AL and CC analyzed the data. PG and AB provided important advice for data interpretation. FF obtained the funding for soil analysis. AL, FF, NDJ, PG, CC, and AB revised the manuscript. All authors contributed to the article and approved the submitted version.

## Conflict of Interest

The authors declare that the research was conducted in the absence of any commercial or financial relationships that could be construed as a potential conflict of interest.

## Publisher’s Note

All claims expressed in this article are solely those of the authors and do not necessarily represent those of their affiliated organizations, or those of the publisher, the editors and the reviewers. Any product that may be evaluated in this article, or claim that may be made by its manufacturer, is not guaranteed or endorsed by the publisher.

## Abbreviations

AD: anaerobic digestate
BBCH: plant phenology scale (Biologische Bundesanstalt, Bundessortenamt, and CHemical industry)
B1: biochar from wood chips (pure)
B1D: biochar from wood chips incubated with digestate
B2: biochar from wheat straw (pure)
B2D: biochar from wheat straw incubated with digestate
DW: dry weight
EC: electrical conductivity
ET: evapotranspiration
FW: fresh weight
PH: plant height
PSA_Licor_: plant shoot area measured with Licor area meter
PSSA: projected shoot system area
SN: spike number
SS: soil substrate
TN: tiller number
WC: plant water content.

## References

[B1] Alburquerque J. A. Salazar P. Barrón V. Torrent J. del Campillo M. C. Gallardo A. (2013). Enhanced wheat yield by biochar addition under different mineral fertilization levels. *Agron. Sustain. Dev.* 33 475–484. 10.1007/s13593-012-0128-3

[B2] Alkharabsheh H. M. Seleiman M. F. Battaglia M. L. Shami A. Jalal R. S. Alhammad B. A. (2021). Biochar and its broad impacts in soil quality and fertility, nutrient leaching and crop productivity: a review. *Agronomy* 11:993. 10.3390/agronomy11050993

[B3] Ayaz M. Feizienë D. Tilvikienë V. Akhtar K. Stulpinaitë U. Iqbal R. (2021). Biochar role in the sustainability of agriculture and environment. *Sustainability* 13:1330. 10.3390/su13031330

[B4] Baronti S. Alberti G. Delle Vedove G. Di Gennaro F. Fellet G. Genesio L. (2010). The biochar option to improve plant yields: first results from some field and pot experiments in Italy. *Ital. J. Agron.* 5 3–11. 10.4081/ija.2010.3

[B5] Bista P. Ghimire R. Machado S. Pritchett L. (2019). Biochar effects on soil properties and wheat biomass vary with fertility management. *Agronomy* 9:623. 10.3390/agronomy9100623

[B6] Celletti S. Lanz M. Bergamo A. Benedetti V. Basso D. Baratieri M. (2021). Evaluating the aqueous phase from hydrothermal carbonization of cow manure digestate as possible fertilizer solution for plant growth. *Front. Plant Sci.* 12:687434. 10.3389/fpls.2021.687434 34276737 PMC8278309

[B7] Chen W. Meng J. Han X. Lan Y. Zhang W. (2019). Past, present, and future of biochar. *Biochar* 1 75–87. 10.1007/s42773-019-00008-3

[B8] Dietrich C. C. Rahaman M. A. Robles-Aguilar A. A. Latif S. Intani K. Müller J. (2020). Nutrient loaded biochar doubled biomass production in juvenile maize plants (*Zea mays* L.). *Agronomy* 10:567. 10.3390/agronomy10040567

[B9] Ding Y. Liu Y. Liu S. Li Z. Tan X. Huang X. (2016). Biochar to improve soil fertility. A review. *Agron. Sustain. Dev.* 23:36. 10.1007/s13593-016-0372-z

[B10] Doyeni M. O. Stulpinaite U. Baksinskaite A. Suproniene S. Tilvikiene V. (2021). The effectiveness of digestate use for fertilization in an agricultural cropping system. *Plants* 10:1734. 10.3390/plants10081734 34451779 PMC8401478

[B11] Fiorani F. Schurr U. (2013). Future scenarios for plant phenotyping. *Annu. Rev. Plant Biol.* 64 267–291. 10.1146/annurev-arplant-050312-120137 23451789

[B12] French E. Iyer-Pascuzzi A. S. (2018). A role for gibberellin pathway in biochar-mediated growth promotion. *Sci. Rep.* 8:5389. 10.1038/s41598-018-23677-9 29599525 PMC5876386

[B13] Gale N. V. Thomas S. C. (2019). Dose-dependence of growth and ecophysiological responses of plants to biochar. *Sci. Tot. Environ.* 658 1344–1354. 10.1016/j.scitotenv.2018.12.239 30677995

[B14] Golzarian M. R. Frick R. A. Rajendran K. Berger B. Roy S. Tester M. (2011). Accurate inference of shoot biomass from high-throughput images of cereal plants. *Plant Methods* 7:2. 10.1186/1746-4811-7-2 21284859 PMC3042986

[B15] Grant C. Cubadda F. Carcea M. Pogna N. E. Gazza L. (2012). “Chapter 7 – Vitamins, minerals, and nutritional value of durum wheat,” in *American Associate of Cereal Chemist International, Durum Wheat*, Second Edn, eds Sissons M. Abecassis J. Marchylo B. Carcea M. (Washington, DC: AACC International Press), 125–137. 10.1016/B978-1-891127-65-6.50012-X

[B16] Grillo F. Piccoli I. Furlanetto I. Ragazzi F. Obber S. Bonato T. (2021). Agro-environmental sustainability of anaerobic digestate fractions in intensive cropping systems: insights regarding the nitrogen use efficiency and crop performance. *Agronomy* 11:745. 10.3390/agronomy11040745

[B17] Hassan M. Liu Y. Naidu R. Parikh S. J. Du J. Qi F. (2020). Influences of feedstock sources and pyrolysis temperature on the properties of biochar and functionality as adsorbents: a meta-analysis. *Sci. Tot. Environ.* 744:140714. 10.1016/j.scitotenv.2020.140714 32717463

[B18] Horie T. Yajima M. Nakagawa H. (1992). Yield forecasting. *Agric. Syst.* 40 211–236. 10.1016/0308-521X(92)90022-G

[B19] Ippolito J. A. Spokas K. A. Novak J. M. Lentz R. D. Cantrell K. B. (2015). “Biochar elemental composition and factors influencing nutrient retention,” in *Biochar for Environmental Management: Science, Technology and Implementation*, 2nd Edn, eds Lehmann J. Joseph S. (New York, NY: Routledge), 137–161.

[B20] Kammann C. I. Schmidt H. P. Messerchmidt N. Linsel S. Steffens D. Müller C. (2015). Plant growth improvement mediated by nitrate capture in co-composted biochar. *Sci. Rep.* 5:11080. 10.1038/srep11080 26057083 PMC4460888

[B21] Laird D. A. (2008). The charcoal vision: a win-win-win scenario for simultaneously producing bioenergy, permanently sequestering carbon, while improving soil and water quality. *Agron. J.* 100 178–181. 10.2134/agronj2007.0161

[B22] Lam V. P. Kim S. J. Park J. S. (2020). Optimizing the electrical conductivity of a nutrient solution for plant growth and bioactive compounds of *Agastache rugosa* in a plant factory. *Agronomy* 10:76. 10.3390/agronomy10010076

[B23] Latini A. Bacci G. Teodoro M. Mirabile Gattia D. Bevivino A. Trakal L. (2019). The impact of soil-applied biochars from different vegetal feedstocks on durum wheat plant performance and rhizospheric bacterial microbiota in low metal-contaminated soil. *Front. Microbiol.* 10:2694. 10.3389/fmicb.2019.0269 31920998 PMC6916200

[B24] Latini A. Giagnacovo G. Campiotti C. A. Bibbiani C. Mariani S. (2021). A narrative review of the facts and perspectives on agricultural fertilization in Europe, with a focus on Italy. *Horticulturae* 7:158. 10.3390/horticulturae7060158

[B25] Lehmann J. (2007). Bio-energy in the black. *Front. Ecol. Environ.* 5 381–387. 10.1890/1540-929520075[381:BITB]2.0.CO;2

[B26] Lei O. Zhang R. (2013). Effects of biochar derived from different feedstockand pyrolysis temperatures on soil, physical and hydraulic properties. *J. Soils Sediments* 13 1561–1572. 10.1007/s11368-013-0738-7

[B27] Li Z. Zhang R. Xia S. Wang L. Liu C. Zhang R. (2019). Interactions between N, P and K fertilizers affect the environment and the yield and quality of satsumas. *Global Ecol.Conserv.* 19:e00663. 10.1016/j.gecco.2019.e00663

[B28] Liu M. Lin Z. Ke X. Fan X. Joseph S. Taherymoosavi S. (2021). Rice seedling growth promotion by biochar varies with genotypes and application dosages. *Front. Plant Sci.* 12:580462. 10.3389/fpls.2021.580462 34234791 PMC8256797

[B29] Meier U. (1997). *BBCH-Monograph. Growth Stages of Plants – Entwicklungsstadien von Pflanzen – Estadios de las Plantas – Développement des Plantes.* Berlin: Blackwell Wissenschafsverlag, 622.

[B30] Nabel M. Schrey S. D. Poorter H. Koller R. Jablonowski N. D. (2017). Effects of digestate fertilization on *Sida hermaphrodita*: boosting biomass yields on marginal soils by increasing soil fertility. *Biomass Bioenergy* 107 207–213. 10.1016/j.biombioe.2017.10.009

[B31] Nakhforoosh A. Bodewein T. Fiorani F. Bodner G. (2016). Identification of water use strategies at early growth stage in durum wheat from shoot phenotyping and physiological measurements. *Front. Plant Sci.* 7:1155. 10.3389/fpls.2016.01155 27547208 PMC4974299

[B32] Nkoa R. (2014). Agricultural benefits and environmental risks of soil fertilization with anaerobic digestates: a review. *Agron. Sustain. Dev.* 34 473–492. 10.1007/s13593-013-0196-z

[B33] Okareh O. T. Gbadebo A. O. (2020). “Enhancement of soil health using biochar. Chapter 9,” in *Applications of Biochar for Environmental Safety*, eds Abdelhafez A. A. Abbas M. H. H. (London: IntechOpen), 10.5772/intechopen.92711

[B34] Pastorelli R. Valboa G. Lagomarsino A. Fabiani A. Simoncini S. Zaghi M. (2021). Recycling biogas digestate from energy crops: effects on soil properties and crop productivity. *Appl. Sci.* 11:750. 10.3390/app11020750

[B35] Pieruschka R. Schurr U. (2019). Plant phenotyping: past, present, and future. *Plant Phenomics* 2019:7507131. 10.34133/2019/7507131 33313536 PMC7718630

[B36] Purakayastha T. J. Bera T. Bhaduri D. Sarkar B. Mandal S. Wade P. (2019). A review on biochar modulated soil condition improvements and nutrient dynamics concerning crop yields: pathways to climate change mitigation and global food security. *Chemosphere* 227 345–365. 10.1016/j.chemosphere.2019.03.170 30999175

[B37] Racioppi M. Tartaglia M. De la Rosa J. M. Marra M. Lopez-Capel E. Roco M. (2019). Response of ancient and modern wheat varieties to biochar application: effect on hormone and gene expression involved in germination and growth. *Agronomy* 10:5. 10.3390/agronomy10010005

[B38] Robles-Aguilar A. A. Temperton V. M. Jablonowski N. D. (2019). Maize silage digestate application affecting germination and early growth of maize modulated by soil type. *Agronomy* 9:473. 10.3390/agronomy9080473

[B39] Ronga D. Caradonia F. Parisi M. Bezzi G. Parisi B. Allesina G. (2020). Using digestate and biochar as fertilizers to improve processing tomato production sustainability. *Agronomy* 10:138. 10.3390/agronomy10010138

[B40] Roy R. Núñez-Delgado A. Sultana S. Wang J. Munir A. Battaglia M. L. (2021). Additions of optimum water, spent mushroom compost and wood biochar to improve the growth performance of *Althaea rosea* in drought-prone coal-mined spoils. *J. Environ. Manage.* 295:113076. 10.1016/j.jenvman.2021.113076 34153587

[B41] Sakhiya A. K. Anand A. Kaushal P. (2020). Production, activation and application of biochar in recent times. *Biochar* 2 253–285. 10.1007/s42773-020-00047-1

[B42] Scharr H. Briese C. Embgenbroich P. Fischbach A. Fiorani F. Müller-Linow M. (2017). Fast high resolution volume carving for 3D plant shoot reconstruction. *Front. Plant Sci.* 8:1680. 10.3389/fpls.2017.01680 29033961 PMC5625571

[B43] Shaaban M. Van Zwieten L. Bashir S. Younas A. Núñez-Delgado A. Chhajro M. A. (2018). A coincise review of biochar application to agricultural soils to improve soil conditions and fight pollution. *J. Environ. Manag.* 228 429–440. 10.1016/j.jenvman.2018.09.006 30243078

[B44] Shahzad K. Abid M. Sintim H. Y. Hussanin S. Nasim W. (2019). Tillage and biochar effects on wheat productivity under arid conditions. *Crop Sci.* 59 1191–1199. 10.2135/cropsci2018.08.0485 34798789

[B45] Sial T. A. Lan Z. Wang L. Zhao Y. Zhang J. Kumbhar F. (2019). Effects of different biochars on wheat growth parameters, yield and soil fertility status in a silty clay loam soil. *Molecules* 24:1798. 10.3390/molecules24091798 31075937 PMC6540089

[B46] Tambone F. Scaglia B. D’Imporzano G. Schievano A. Orzi V. Salati S. (2010). Assessing amendment and fertilizing properties of digestates from anaerobic digestion through a comparative study with digested sludge and compost. *Chemosphere* 81 577–583. 10.1016/j.chemosphere.2010.08.034 20825964

[B47] Tomczyk A. Sokolowska Z. Boguta P. (2020). Biochar physicochemical properties: pyrolysis temperature and feedstock kind effects. *Rev. Environ. Sci. Biotechnol.* 19 191–215. 10.1007/s11157-020-09523-3

[B48] Vaccari F. P. Baronti S. Lugato E. Genesio L. Castaldi S. Fornasier F. (2011). Biochar as a strategy to sequester carbon and increase yield in durum wheat. *Eur. J.Agron.* 34 231–238. 10.1016/j.eja.2011.01.006

[B49] VDLUFA (1991). *Methodenbuch Band I. Die Untersuchung Von Böden.* Darmstadt: VDLUFA-Verlag.

[B50] Vijay V. Shreedhar S. Adlak K. Payyanad S. Sreedharan V. Gopi G. (2021). Review of large-scale biochar field-trials for soil amendment and the observed influences on crop yield variations. *Front. Energy Res.* 9:710766. 10.3389/fenrg.2021.710766

[B51] Walsh J. J. Jones D. L. Edwards-Jones G. Williams A. P. (2012). Replacing inorganic fertilizer with anaerobic digestate may maintain agricultural productivity at less environmental cost. *J. Plant Nutr. Soil Sci.* 175 840–845. 10.1002/jpln.201200214

[B52] Win K. T. Okazaki K. Ookawa T. Yokoyama T. Ohwaki Y. (2019). Influence of rice-husk biochar and *Bacillus pumilus* strain TUAT-1 on yield, biomass production, and nutrient uptake in two forage rice genotypes. *PLoS One* 14:e0220236. 10.1371/journal.pone.0220236 31365570 PMC6668810

[B53] Yaashikaa P. R. Kumar P. S. Varjani S. Saravanan A. (2020). A critical review on the biochar production techniques, characterization, stability and applications for circular bioeconomy. *Biotechnol. Rep.* 28:e00570. 10.1016/j.btre.2020.e00570 33304842 PMC7718465

[B54] Zhao L. Cao X. Mašek O. Zimmerman A. (2013). Heterogeneity of biochar properties as a function of feedstock sources and production temperatures. *J. Haz. Mat.* 256–257 1–9. 10.1016/j.jhazmat.2013.04.015 23669784

