# Supplementary material for: Phenotyping of Different Italian Durum Wheat Varieties in Early Growth Stage With the Addition of Pure or Digestate-Activated Biochars
Source: Front Plant Sci. 2021 Dec 20;12:782072. doi: 10.3389/fpls.2021.782072 (PMC8721205; doi:10.3389/fpls.2021.782072)
Supplement: Supplementary file 1 [file Data_Sheet_1.docx]

Supplementary Material 1

**BBCH statistical data analysis**

The phenological developmental stage was evaluated by BBCH two times during the experiment, at day 49 (7 weeks) and at day 56 (almost end of the experiment, *i.e*. day 58). The statistical analysis has been conducted with SPSS software (IBM). Data showed different and sometimes high standard deviations, leading to the significance of the Levene test on the homogeneity of variance (Sig. 0.00) and of the Shapiro-Wilk test on the normality of data (Sig. <0.05). Since the assumptions of the equality of variance and the normal data distribution were not met, the two-way ANOVA could not be used as statistical test; anyway, it did not reveal any interaction effect between the factors genotype and treatment. Consequently, data was analysed by one-way ANOVA, Welch (Sig. 0.00) and Games-Howell as *post-hoc* test.

*One-way ANOVA*

Using “genotype” as factor, there were statistically significant differences between group means in both BBCH datasets (F(4,145) = 14.224, p = 0.000 for BBCH_day49_, and F(4,145) = 15.440, p = 0.000 for BBCH_day56_).

Using “treatment” as factor, there were statistically significant differences between group means in both BBCH datasets (F(4,145) = 15.006, p = 0.000 for BBCH_day49_, and F(4,145) = 11.516, p = 0.000 for BBCH_day56_).

*Post-hoc analysis*

Games-Howell outputs and the mean plot – directly exported from SPSS - with the BBCH data estimated after 7 weeks from seed germination, using “genotype” as factor, are reported below:

| **Multiple Comparisons** | | | | | | |
| --- | --- | --- | --- | --- | --- | --- |
| Dependent Variable: BBCH_day49_ | | | | | | |
| Games-Howell | | | | | | |
| (I) GENOTYPE | (J) GENOTYPE | Mean Difference (I-J) | Std. Error | Sig. | 95% Confidence Interval | |
|  |  |  |  |  | Lower Bound | Upper Bound |
| Duilio | Grecale | -5,13333* | 1,56751 | ,015 | -9,5468 | -,7198 |
|  | Iride | 4,50000* | 1,39007 | ,017 | ,5805 | 8,4195 |
|  | Marco Aurelio | -6,10000* | 1,50993 | ,001 | -10,3512 | -1,8488 |
|  | Saragolla | -,40000 | 1,81330 | ,999 | -5,5185 | 4,7185 |
| Grecale | Duilio | 5,13333* | 1,56751 | ,015 | ,7198 | 9,5468 |
|  | Iride | 9,63333* | 1,42803 | ,000 | 5,6043 | 13,6623 |
|  | Marco Aurelio | -,96667 | 1,54495 | ,970 | -5,3171 | 3,3838 |
|  | Saragolla | 4,73333 | 1,84256 | ,091 | -,4642 | 9,9308 |
| Iride | Duilio | -4,50000* | 1,39007 | ,017 | -8,4195 | -,5805 |
|  | Grecale | -9,63333* | 1,42803 | ,000 | -13,6623 | -5,6043 |
|  | Marco Aurelio | -10,60000* | 1,36458 | ,000 | -14,4461 | -6,7539 |
|  | Saragolla | -4,90000* | 1,69418 | ,044 | -9,7038 | -,0962 |
| Marco Aurelio | Duilio | 6,10000* | 1,50993 | ,001 | 1,8488 | 10,3512 |
|  | Grecale | ,96667 | 1,54495 | ,970 | -3,3838 | 5,3171 |
|  | Iride | 10,60000* | 1,36458 | ,000 | 6,7539 | 14,4461 |
|  | Saragolla | 5,70000* | 1,79383 | ,020 | ,6338 | 10,7662 |
| Saragolla | Duilio | ,40000 | 1,81330 | ,999 | -4,7185 | 5,5185 |
|  | Grecale | -4,73333 | 1,84256 | ,091 | -9,9308 | ,4642 |
|  | Iride | 4,90000* | 1,69418 | ,044 | ,0962 | 9,7038 |
|  | Marco Aurelio | -5,70000* | 1,79383 | ,020 | -10,7662 | -,6338 |

*. The mean difference is significant at the 0.05 level.

**Plot Means**


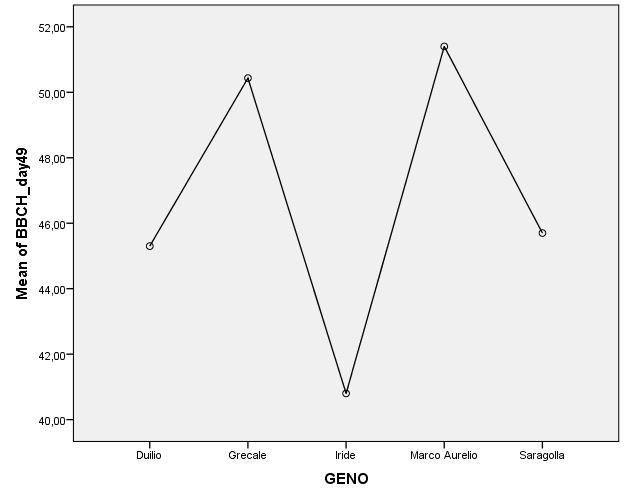


Games-Howell outputs and the mean plot – directly exported from SPSS – with the BBCH data estimated after 7 weeks from seed germination, using “genotype” as factor, are reported below:

| **Multiple Comparisons** | | | | | | |
| --- | --- | --- | --- | --- | --- | --- |
| Dependent Variable: BBCH_day56_ | | | | | | |
| Games-Howell | | | | | | |
| (I) GENOTYPE | (J) GENOTYPE | Mean Difference (I-J) | Std. Error | Sig. | 95% Confidence Interval | |
|  |  |  |  |  | Lower Bound | Upper Bound |
| Duilio | Grecale | -5,467^*^ | 1,111 | ,000 | -8,62 | -2,31 |
|  | Iride | 2,967 | 1,316 | ,175 | -,74 | 6,67 |
|  | Marco Aurelio | -2,733 | 1,125 | ,126 | -5,93 | ,46 |
|  | Saragolla | ,333 | 1,430 | ,999 | -3,69 | 4,36 |
| Grecale | Duilio | 5,467^*^ | 1,111 | ,000 | 2,31 | 8,62 |
|  | Iride | 8,433^*^ | 1,035 | ,000 | 5,50 | 11,37 |
|  | Marco Aurelio | 2,733^*^ | ,779 | ,008 | ,54 | 4,93 |
|  | Saragolla | 5,800^*^ | 1,177 | ,000 | 2,45 | 9,15 |
| Iride | Duilio | -2,967 | 1,316 | ,175 | -6,67 | ,74 |
|  | Grecale | -8,433^*^ | 1,035 | ,000 | -11,37 | -5,50 |
|  | Marco Aurelio | -5,700^*^ | 1,050 | ,000 | -8,67 | -2,73 |
|  | Saragolla | -2,633 | 1,372 | ,319 | -6,50 | 1,23 |
| Marco Aurelio | Duilio | 2,733 | 1,125 | ,126 | -,46 | 5,93 |
|  | Grecale | -2,733^*^ | ,779 | ,008 | -4,93 | -,54 |
|  | Iride | 5,700^*^ | 1,050 | ,000 | 2,73 | 8,67 |
|  | Saragolla | 3,067 | 1,190 | ,092 | -,32 | 6,45 |
| Saragolla | Duilio | -,333 | 1,430 | ,999 | -4,36 | 3,69 |
|  | Grecale | -5,800^*^ | 1,177 | ,000 | -9,15 | -2,45 |
|  | Iride | 2,633 | 1,372 | ,319 | -1,23 | 6,50 |
|  | Marco Aurelio | -3,067 | 1,190 | ,092 | -6,45 | ,32 |
| *. The mean difference is significant at the 0.05 level. | | | | | | |

**Plot Means**


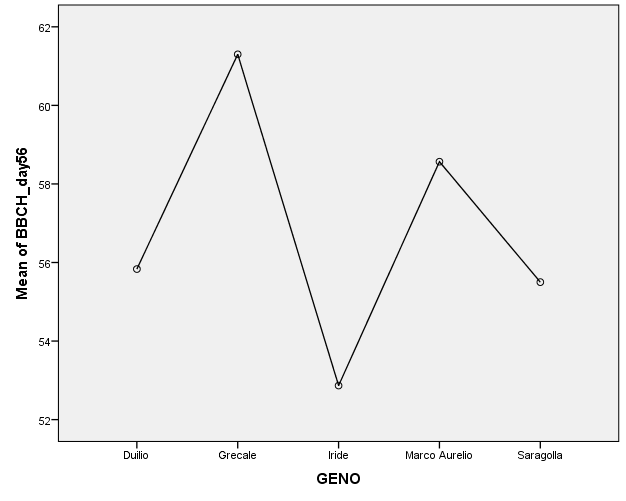


Games-Howell outputs and the mean plot – directly exported from SPSS – with the BBCH data estimated after 7 weeks from seed germination, using “treatment” as factor, are reported below:

| **Multiple Comparisons** | | | | | | |  |
| --- | --- | --- | --- | --- | --- | --- | --- |
| Dependent Variable: BBCH_day49_ | | | | | | |  |
| Games-Howell | | | | | | |  |
| (I) TREATMENT | (J) TREATMENT | Mean Difference (I-J) | Std. Error | Sig. | 95% Confidence Interval | | |
|  |  |  |  |  | Lower Bound | Upper Bound | |
| C- | B1 | -1,26667 | 1,67149 | ,941 | -5,9833 | 3,4499 | |
|  | B1D | -,53333 | 1,43892 | ,996 | -4,5845 | 3,5179 | |
|  | B2 | -9,53333^*^ | 1,55930 | ,000 | -13,9269 | -5,1398 | |
|  | B2D | -7,63333^*^ | 1,42443 | ,000 | -11,6437 | -3,6229 | |
| B1 | C- | 1,26667 | 1,67149 | ,941 | -3,4499 | 5,9833 | |
|  | B1D | ,73333 | 1,67753 | ,992 | -3,9996 | 5,4662 | |
|  | B2 | -8,26667^*^ | 1,78186 | ,000 | -13,2855 | -3,2479 | |
|  | B2D | -6,36667^*^ | 1,66511 | ,003 | -11,0661 | -1,6673 | |
| B1D | C- | ,53333 | 1,43892 | ,996 | -3,5179 | 4,5845 | |
|  | B1 | -,73333 | 1,67753 | ,992 | -5,4662 | 3,9996 | |
|  | B2 | -9,00000^*^ | 1,56577 | ,000 | -13,4114 | -4,5886 | |
|  | B2D | -7,10000^*^ | 1,43151 | ,000 | -11,1304 | -3,0696 | |
| B2 | C- | 9,53333^*^ | 1,55930 | ,000 | 5,1398 | 13,9269 | |
|  | B1 | 8,26667^*^ | 1,78186 | ,000 | 3,2479 | 13,2855 | |
|  | B1D | 9,00000^*^ | 1,56577 | ,000 | 4,5886 | 13,4114 | |
|  | B2D | 1,90000 | 1,55247 | ,738 | -2,4748 | 6,2748 | |
| B2D | C- | 7,63333^*^ | 1,42443 | ,000 | 3,6229 | 11,6437 | |
|  | B1 | 6,36667^*^ | 1,66511 | ,003 | 1,6673 | 11,0661 | |
|  | B1D | 7,10000^*^ | 1,43151 | ,000 | 3,0696 | 11,1304 | |
|  | B2 | -1,90000 | 1,55247 | ,738 | -6,2748 | 2,4748 | |

*. The mean difference is significant at the 0.05 level.

**Plot Means**


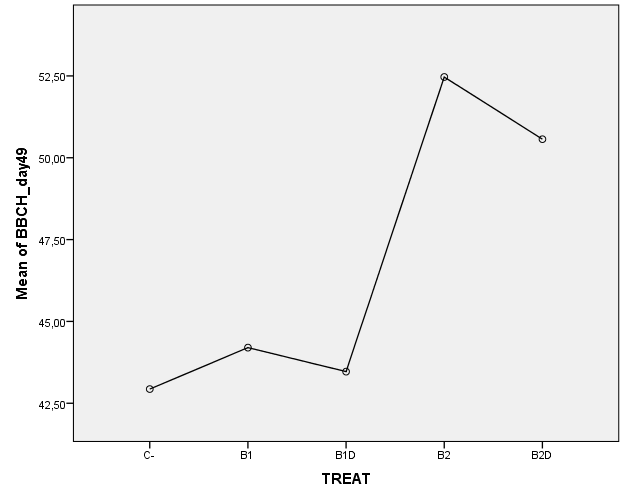


Games-Howell outputs and the mean plot – directly exported from SPSS – with the BBCH data estimated at the end of the experiment, using “treatment” as factor, are reported below:

| **Multiple Comparisons** | | | | | | |  |
| --- | --- | --- | --- | --- | --- | --- | --- |
| Dependent Variable: BBCH_day56_ | | | | | | |  |
| Games-Howell | | | | | | |  |
| (I) TREATMENT | (J) TREATMENT | Mean Difference (I-J) | Std. Error | Sig. | 95% Confidence Interval | | |
|  |  |  |  |  | Lower Bound | Upper Bound | |
| C- | B1 | 1,467 | 1,479 | ,858 | -2,71 | 5,65 | |
|  | B1D | -,433 | 1,219 | ,996 | -3,86 | 3,00 | |
|  | B2 | -5,200* | 1,025 | ,000 | -8,10 | -2,30 | |
|  | B2D | -4,733* | 1,051 | ,000 | -7,70 | -1,77 | |
| B1 | C- | -1,467 | 1,479 | ,858 | -5,65 | 2,71 | |
|  | B1D | -1,900 | 1,485 | ,705 | -6,09 | 2,29 | |
|  | B2 | -6,667* | 1,331 | ,000 | -10,46 | -2,87 | |
|  | B2D | -6,200* | 1,350 | ,000 | -10,05 | -2,35 | |
| B1D | C- | ,433 | 1,219 | ,996 | -3,00 | 3,86 | |
|  | B1 | 1,900 | 1,485 | ,705 | -2,29 | 6,09 | |
|  | B2 | -4,767* | 1,033 | ,000 | -7,69 | -1,84 | |
|  | B2D | -4,300* | 1,059 | ,001 | -7,29 | -1,31 | |
| B2 | C- | 5,200* | 1,025 | ,000 | 2,30 | 8,10 | |
|  | B1 | 6,667* | 1,331 | ,000 | 2,87 | 10,46 | |
|  | B1D | 4,767* | 1,033 | ,000 | 1,84 | 7,69 | |
|  | B2D | ,467 | ,828 | ,980 | -1,87 | 2,80 | |
| B2D | C- | 4,733* | 1,051 | ,000 | 1,77 | 7,70 | |
|  | B1 | 6,200* | 1,350 | ,000 | 2,35 | 10,05 | |
|  | B1D | 4,300* | 1,059 | ,001 | 1,31 | 7,29 | |
|  | B2 | -,467 | ,828 | ,980 | -2,80 | 1,87 | |

*. The mean difference is significant at the 0.05 level.

**Plot Means**


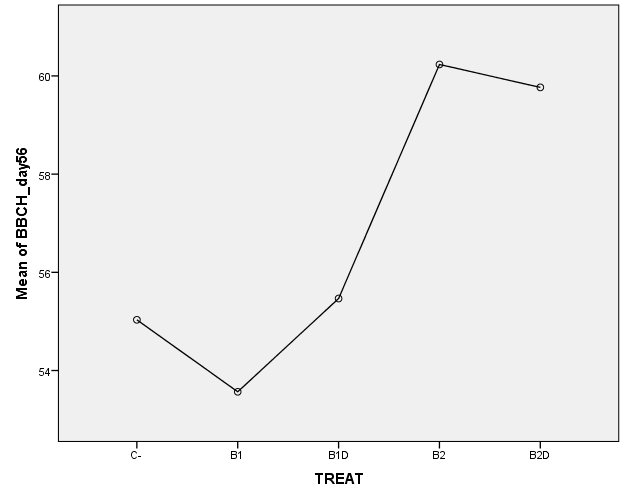


Using “genotype” as categorical variable, at 7 weeks, Grecale (50.43±6.202) and Marco Aurelio (51.40±5.757) showed the highest BBCH values, Iride the lowest one (40.80±4.766), Duilio (45.30±5.937) and Saragolla (45.70±7.962) in the between. Similarly, close to the end of the experiment, Grecale (61.30±2.938) and Marco Aurelio (58.57±3.093) showed the highest BBCH values, Iride the lowest one (52.87±4.848), Duilio (55.83±5.331) and Saragolla (55.50±5.740) in the between.

Using “treatment” as categorical variable, at 7 weeks, B2 (52.47±6.495) and B2D (50.57±5.488) showed the highest BBCH values, while C- (42.93±5.546), B1 (44.20±7.284), and B1D (43.47±5.600) showed lower and similar values. Similarly, close to the end of the experiment, B2 (60.23±3.081) and B2D (59.77±3.329) showed the highest values. C- (55.03±4.694), B1 (53.57±6.606), and B1D (55.47±4.747) showed lower values compared with B2 and B2D, but similar values among each other.

*In summary:*

With “genotype” as categorical variable, Marco Aurelio and Grecale showed the highest BBCH values, Iride the lowest, while the values for Duilio and Saragolla were in between. With “treatment” as categorical variable, B2 and B2D showed the highest BBCH values. C-, B1, and B1D showed lower values compared with B2 and B2D, but similar values among each other.
