# Supplementary material for: Phenotyping of Different Italian Durum Wheat Varieties in Early Growth Stage With the Addition of Pure or Digestate-Activated Biochars
Source: Front Plant Sci. 2021 Dec 20;12:782072. doi: 10.3389/fpls.2021.782072 (PMC8721205; doi:10.3389/fpls.2021.782072)
Supplement: Supplementary file 2 [file Data_Sheet_2.PDF]

frontiers

Supplementary Material 2

Statistical analysis of the shoot plant area at the end of the experiment

Assumption of normality distribution of data

Plant area has been estimated with two parameters: the projected shoot system area by RBG image analysis (PSSA at day 56) and plant shoot area by Licor area meter at T_f (PSA_{Licor}). Besides the Shapiro-Wilk test, the normality of data has been investigated through Skewness and Kurtosis. For normality, the ratio between Skewness value and its standard error, and the ratio between Kurtosis value and its standard error should be |2| (George and Mallery 2010)[1]. As evidenced in blue bold, PSSA deviated from normality according to the Shapiro-Wilk ($p \leq 0.05$) and the Skewness (ratio = 2.722) tests; on the other side, PSA_{Licor} data distributed normally.

	Shapiro-Wilk			Skewness			Kurtosis		
	Statistic	df	Sig.	Statistic	Std. error	ratio	Statistic	Std. error	ratio.
PSSA	0.966	150	**0.001**	-0.539	0.198	**2.722**	-0.088	0.394	0.223
PSA_{Licor}	0.986	150	0.127	-0.168	0.198	0.848	-0.098	0.394	0.249

Abbreviations: projected shoot system area (PSSA), total plant shoot area by Licor area meter (PSA_{Licor})

Consequently, for PSSA, the hypothesis of the normality of data was examined more in depth conducting the above tests for each level of the two independent variables (5 genotypes and 5 treatments). As a result, the violation from the normality of data was related exclusively to the genotype Grecale (Shapiro-Wilk p=0.001 and Skewness ratio > 2, in blue bold).

		Shapiro-Wilk			Skewness			Kurtosis		
		Statistic	df	Sig.	Statistic	Std. error	ratio	Statistic	Std. error	ratio
Independent variable “genotype”	Duilio	0.932	30	0.054	-0.038	0.427	0.089	-1.217	0.833	1.461
	Grecale	0.857	30	**0.001**	-0.876	0.427	**2.052**	-0.630	0.833	0.756
	Iride	0.954	30	0.215	-0.374	0.427	0.876	-0.788	0.833	0.946
	Marco Aurelio	0.941	30	0.099	-0.095	0.427	0.222	-1.126	0.833	1.352
	Saragolla	0.956	30	0.240	-0.308	0.427	0.721	-0.767	0.833	0.921
Independent variable “treatment”	C-	0.952	30	0.190	-0.097	0.427	0.227	-0.993	0.833	1.192
	B1	0.952	30	0.189	0.715	0.427	1.674	1.191	0.833	1.430
	B1D	0.981	30	0.864	0.317	0.427	0.742	0.122	0.833	0.146
	B2	0.932	30	0.055	0.019	0.427	0.044	-1.403	0.833	1.684
	B2D	0.963	30	0.370	-0.231	0.427	0.541	-0.148	0.833	0.178

Abbreviations: soil substrate (SS) lacking any biochar treatment (C-); biochar from wood chips (B1); B1 incubated with digestate (B1D); biochar from wheat straw (B2); B2 incubated with digestate (B2D)

[1]George D, Mallery M (2010) SPSS for Windows Step by Step: A Simple Guide and Reference. 17.0 Update. 10th Ed. Allyn & Bacon. ISBN 0205755615

Assumption of equality of variance

The Levene test for both PSSA and PSA_{Licor} data resulted in the violation of the assumption of the equality of variance (p<0.05 in blue bold). For PSSA, since data did not show a normal distribution, it was also used a non-parametric Levene test, with p>0.05 indicating that the homoscedasticity requirement is met (Nordstokke and Zumbo 2010[2]; Nordstokke et al. 2011[3]), but the assumption of equality of variance was validated only when using “genotype” as categorical variable (p=0.249).

	Parametric Levene test				Nonparametric Levene test							
					with “genotype” as factor				with “treatment” as factor			
	F	df1	df2	Sig.	Levene statistic	df1	df2	Sig.	Levene statistic	df1	df2	Sig.
PSSA	2.050	24	125	0.006	1.365	4	145	0.249	5.320	4	145	0.001
PSA	2.085	24	125	**0.005**								

Abbreviations: projected shoot system area (PSSA), total plant shoot area by Licor area meter (PSA_{Licor})

Two-way ANOVA

The two-way ANOVA has been used to compare the mean differences between groups, with the objective to evidence if there is any interaction between the plant shoot area (dependent variable) and the genotype and the treatment (independent variables). The analysis performed followed the suggestions from Kim (2014)[4]. Each of the two fixed factors presented 5 levels (5 genotypes and 5 treatments). Even though the two main assumptions of normality and equality of variance were not perfectly matched for PSSA and PSA_{Licor} data, this test is considered very robust, particularly thanks to the large sample size; moreover, the significance level was reduced to 0.01, thus increasing the confidence of the results (99%).

The relationship between the two categorical variables and plant shoot area, for PSSA and for PSA_{Licor}, is schematically shown in the mean plot, where an additive relationship between the two independent variables (interaction model) is evident for the treatments C-, B1 and B1D

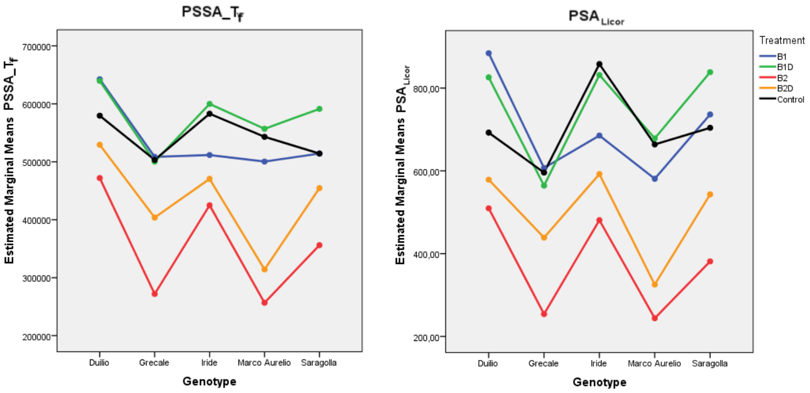

[2]Nordstokke DW, Zumbo BD (2010) A new nonparametric Levene test for equal variances. Psicológica 31:401-430

[3]Nordstokke DW, Zumbo BD, Cairns SL, Saklofske DH (2011) The operating characteristics of the non parametric Levene test for equal variances with assessment and evaluation data. Practical Assess Res Eval 16:5. https://doi.org/10.7275/5t99-zv93

[4]Kim HY (2014) Statistical notes for clinical researchers: two-way analysis of variance (ANOVA)-exploring possible interaction between factors. Restor Dent Endod 39:143-147. https://doi.org/10.5395/rde.2014.39.2.143

The results of the two-way ANOVA are reported in Table 5 of the manuscript. Briefly, there was a significant effect on the data due to the genotype and to the treatment ($p<0.001$ for both PSSA and PSA_{Licor}), while the interaction term "genotype*treatment" was statistically significant ($p<0.01$) for PSSA, but not significant ($p>0.05$) for PSA_{Licor}.

The table below reports the descriptive statistics results (mean ± SD) from the interaction model and the *post-hoc* multiple comparisons.

		Treatment				
	Genotype	Control	B1	B1D	B2	B2D
PSSA	Duilio	579547.67 ± 73207.825 **Aa**	642460.00 ± 77132.272 **Aa**	639663.83 ± 35527.125 **Aa**	471991.17 ± 21099.392 **Ac**	529403.17 ± 83102.443 **Ab**
	Grecale	503351.33 ± 54817.979 **Ca**	508511.00 ± 17494.304 **Ca**	500184.00 ± 45652.682 **Ca**	271842.17 ± 55529.533 **Cc**	403718.17 ± 113460.650 **Cb**
	Iride	582949.50 ± 25056.325 **ABa**	511698.67 ± 66080.205 **ABa**	599874.50 ± 30486.678 **ABa**	425004.83 ± 52023.055 **ABc**	470527.67 ± 39242.684 **ABb**
	Marco Aurelio	543137.67 ± 61324,874 **Ca**	500386.00 ± 64065.737 **Ca**	556941.50 ± 88363.743 **Ca**	256738.00 ± 39394.801 **Cc**	314220.50 ± 53255.282 **Cb**
	Saragolla	514231.67 ± 61500.145 **BCa**	514151.67 ± 64709.528 **BCa**	591295.50 ± 42225.420 **BCa**	356113.67 ± 53319.123 **BCc**	454522.67 ± 49525.323 **BCb**
PSA_{Licor}	Duilio	692.44 ± 140.315 **Aa**	884.25 ± 260.782 **Aa**	825.95± 109.913 **Aa**	509.54 ± 25.077 **Ac**	578.68 ± 123.300 **Ab**
	Grecale	595.81 ± 81.035 **Ba**	606.96 ± 58.237 **Ba**	564.31 ± 76.126 **Ba**	254.07 ± 64.458 **Bc**	438.69 ± 155.352 **Bb**
	Iride	858.00 ± 90.278 **Aa**	685.31 ± 115.334 **Aa**	831.61 ± 78.283 **Aa**	480.92 ± 83.052 **Ac**	592.29 ± 68.764 **Ab**
	Marco Aurelio	663.84 ± 131.147 **Ba**	580.92 ± 106.227 **Ba**	678.45 ± 155.439 **Ba**	243.95 ± 48.847 **Bc**	325.22 ± 71.898 **Bb**
	Saragolla	703.96 ± 171.061 **Aa**	736.33 ± 174.975 **Aa**	838.46 ± 125.455 **Aa**	381.29 ± 92.308 **Ac**	543.16 ± 92.239 **Ab**

Abbreviations: projected shoot system area (PSSA), total plant shoot area by Licor area meter (PSA_{Licor}).

* Different bold letters indicate significant difference according to the R-E-G-W-Q test at $p<0.001$ level, with the black uppercase letters referring to the "genotype" subset and the blue lowercase ones to the "treatment" subset.

R-E-G-W-Q has been used as *post-hoc* test, because it offers a good control on type 1 errors in presence of a similar sample size as in the current data set. The homogeneous subsets resulting for the two dependent variables, PSSA and PSA_{Licor}, are reported below, separately for "genotype" and "treatment".

PSSA dependent variable and "genotype" as independent variable:

GENOTYPE	N	Subset for alpha = 0.001		
		A	B	C
Marco Aurelio	30			434284.73
Grecale	30			437521.33
Saragolla	30		486063.03	486063.03
Iride	30	518011.03	518011.03	
Duilio	30	572613.17		
Sig.		0.001	0.0915	0.004

PSSA dependent variable and "treatment" as independent variable:

TREATMENT	N	Subset for alpha = 0.001		
		a	b	c
B2	30			356337.97
B2D	30		434478.43	
B1	30	535441.47		
C-	30	544643.57		
B1D	30	577591.87		
Sig.		0.029	1.000	1.000

PSA_{Licor} dependent variable and "genotype" as independent variable:

GENOTYPE	N	Subset for alpha = 0.001	
		A	B
Grecale	30		491.969
Marco Aurelio	30		498.475
Saragolla	30	640.638	
Iride	30	689.625	
Duilio	30	698.172	
Sig.		0.236	0.988

PSA_{Licor} dependent variable and "treatment" as independent variable:

TREATMENT	N	Subset for alpha = 0.001		
		a	b	c
B2	30			373.953
B2D	30		495.607	
B1	30	698.753		
C-	30	702.810		
B1D	30	747.755		
Sig.		0.380	1.000	1.000

These results are also reported in the manuscript in the form of the two histograms for PSSA and PSA_{Licor} at the end of the experiment (Fig. 3a and b, respectively).
