# Supplementary material for: Phenotyping of Different Italian Durum Wheat Varieties in Early Growth Stage With the Addition of Pure or Digestate-Activated Biochars
Source: Front Plant Sci. 2021 Dec 20;12:782072. doi: 10.3389/fpls.2021.782072 (PMC8721205; doi:10.3389/fpls.2021.782072)
Supplement: Supplementary file 3 [file Data_Sheet_3.docx]

Supplementary Material 3

**Microclimatic conditions**

The microclimate parameters measured inside the ScreenHouse and analysed throughout the experiment were temperature (T, °C), relative humidity (RH, %) and global radiation (PPDF, µmol·m/s). Six data loggers for each physical parameter were appropriately positioned inside the greenhouse, in proximity of the pots and at the level of the plant canopies, and the set recording interval was one per hour.

Overall, temperature major variations were registered during the warmer hours of the day, mainly from 12:00 to 16:00, with the higher inter-day variation from 27.8 °C in day 2 to 19.2 °C in day 58, at 15:00 (Fig. **a**). According to the annual period (October-November), during approximately the last twenty days of the experiment, the temperature in the warmer hours was around 20 °C, or a little below, as it can be deduced in the Fig. **a** by the light colours of the lines (a different colour/indicator is associated to each day, with the lighter tonalities being related to the last days of the experiment). Average relative humidity showed an intra- and inter-day variation higher than temperature, particularly during the first morning hours and the warmer ones (Fig. **b**). Measured RH levels fell mostly in the range of 60-80%, with the exception of the warmer hours during the first 30-35 days of the experiment, where RH percentages below the 50%, and even below the 40% exclusively at days 3 and 13, were often registered (Fig. **b**). The global radiation measured close to the canopy level has been estimated from the average photosynthetic photon flux density (PPDF), which corresponds to the amount of photosynthetically active photons hitting a surface per unit of area and unit of time. As shown in Fig. **c**, PPDF varied strongly during the daily hours, and the highest values were registered during the first 30-35 days.

Furthermore, the Pearson correlation analysis with the environmental data acquired inside the ScreenHouse evidenced a strong positive correlation between T and PAR (R = 0.826, p<0.01), and moderate negative correlations between RH and T (R = -0.580, p<0.01), and RH and PAR (R = -0.602, p<0.01).


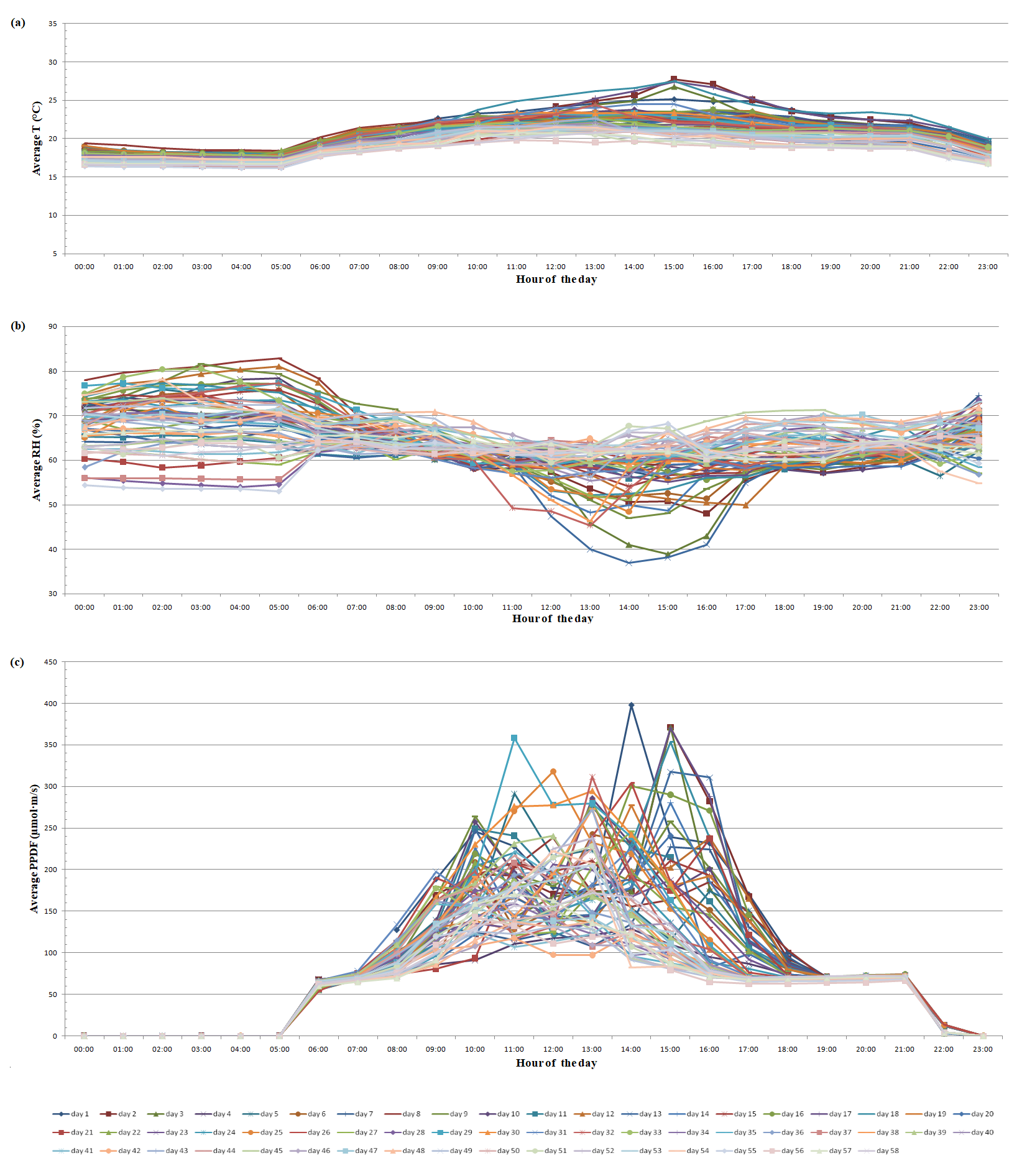


**Supplementary Figure.** Intra- and inter-day trend of average temperature **(a)**, relative humidity **(b)** and photosynthetic photon density flux **(c)** inside the ScreenHouse. For each parameter, measured hourly and throughout the experiment, the values reported in the line graphs correspond to the average of the six readings, each registered by one of the six monitoring sensors.
