# Supplementary material for: Phenotyping of Different Italian Durum Wheat Varieties in Early Growth Stage With the Addition of Pure or Digestate-Activated Biochars
Source: Front Plant Sci. 2021 Dec 20;12:782072. doi: 10.3389/fpls.2021.782072 (PMC8721205; doi:10.3389/fpls.2021.782072)
Supplement: Supplementary file 4 [file Data_Sheet_4.PDF]

Supplementary Material 4

Statistical analysis of the evapotranspiration (ET) at the end of the experiment (day 56)

Assumption of normality distribution of data

The normality of ET_{d56} data has been ascertained by the Shapiro-Wilk test, being Sig. >0.05.

Shapiro-Wilk		
Statistic	df	Sig.
0.987	149	0.178

Assumption of equality of variance

The equality of variances of ET_{d56} data has been verified by the Levene test, being Sig. >0.05.

Parametric Levene test							
with "genotype" as factor				with "treatment" as factor			
Levene statistic	df1	df2	Sig.	Levene statistic	df1	df2	Sig.
1.383	4	144	0.243	1.237	4	144	0.298

Two-way ANOVA

The two-way ANOVA has been used to compare the mean differences between groups, with the objective to evidence if there is any interaction between the ET_{d56} (dependent variable) and the genotype and the treatment (independent variables). The analysis performed followed the suggestions from Kim (2014)[1]. Each of the two fixed factors presented 5 levels (5 genotypes and 5 treatments). This test is considered very robust, particularly thanks to the large sample size; moreover, the significance level was reduced to 0.001, thus increasing the confidence of the results (99.9%).

The results of the two-way ANOVA are reported in the Table 5 of the manuscript. Briefly, there were significant effects on the ET parameter due to the "genotype" and to the "treatment" (p<0.001), while the interaction term "genotype*treatment" was not statistically significant (p>0.05).

The table below reports the descriptive statistics results (mean ± SD) from the interaction model and the post hoc multiple comparisons (see next page).

[1] Kim HY (2014) Statistical notes for clinical researchers: two-way analysis of variance (ANOVA)-exploring possible interaction between factors. Restor Dent Endod 39:143-147. https://doi.org/10.5395/rde.2014.39.2.143

	Treatment				
Genotype	C-	B1	B1D	B2	B2D
Duilio	138.50 ± 27.887 **Aa**	148.83 ± 26.480 **Aab**	154.08 ± 24.899 **Aa**	95,58 ± 23.604 **Ac**	103.50 ± 18.868 **Ab**
Grecale	110.58 ± 21.113 **Ca**	116.83 ± 17.105 **Cab**	111.75 ± 8.993 **Ca**	64.33 ± 5.750 **Cc**	91.50 ± 18.898 **Cb**
Iride	119.00 ± 20.137 **ABCa**	117.67 ± 13.644 **ABCab**	137.17 ± 6.728 **ABCa**	80.33 ± 13.400 **ABCc**	111.42 ± 12.866 **ABCb**
Marco Aurelio	127.00 ± 9.560 **BCa**	113.00 ± 14.680 **BCab**	117.58 ± 19.923 **BCa**	96.08 ± 13.313 **BCc**	96.08 ± 13.313 **BCb**
Saragolla	107.25 ± 16.634 **ABa**	136.08 ± 32.302 **ABab**	148.58 ± 26.828 **ABa**	90.75 ± 18,796 **ABc**	110.67 ± 13.692 **ABb**

Abbreviations: soil substrate (SS) lacking any biochar treatment (C-); biochar from wood chips (B1); B1 incubated with digestate (B1D); biochar from wheat straw (B2); B2 incubated with digestate (B2D).

* Different bold letters indicate significant difference according to the R-E-G-W-Q test at $p<0.001$ level, with black uppercase letters referring to the "genotype" subset and the blue lowercase ones to the "treatment" subset.

R-E-G-W-Q has been used as *post-hoc* test, because it offers a good control of type 1 errors in presence of a similar sample size as in the current data set. The homogeneous subsets resulting for the dependent variable ET_{d56} are reported below, separately for "genotype" and "treatment".

ET_{d56} as dependent variable and "genotype" as independent variable:

GENOTYPE	N	Subset for alpha = 0.001		
		A	B	C
Grecale	30			99.26
Marco Aurelio	30		105.48	105.48
Iride	30	113.12	113.12	113.12
Saragolla	30	118.67	118.67	
Duilio	30	128.10		
Sig.		0.011	0.033	0.026

ET_{d56} as dependent variable and "treatment" as independent variable:

TREATMENT	N	Subset for alpha = 0.001		
		a	b	c
B2	30			80.95
B2D	30		103.02	
B1	30	120.47	120.47	
C-	30	126.48		
B1D	30	133.83		
Sig.		0.030	0.001	1.000
