# Supplementary material for: Phenotyping of Different Italian Durum Wheat Varieties in Early Growth Stage With the Addition of Pure or Digestate-Activated Biochars
Source: Front Plant Sci. 2021 Dec 20;12:782072. doi: 10.3389/fpls.2021.782072 (PMC8721205; doi:10.3389/fpls.2021.782072)
Supplement: Supplementary file 5 [file Data_Sheet_5.PDF]

Supplementary Material 5

Correlation analysis between evapotranspiration (ET) and projected shoot system area (PSSA) time-series datasets

The scatterplot below displays the values of ET and PSSA with the line of fit by linear regression **(a)**, by quadratic regression **(b)**, and by LOESS (LOcally WEighted Scatter-plot Smoother) curve fitting-local regression **(c)**.

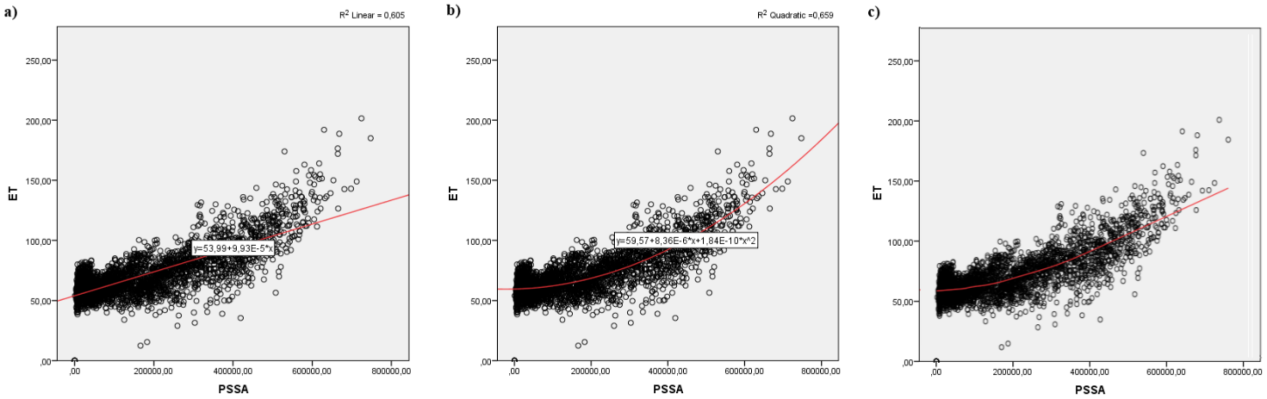

The data distribution tends to a non-linear correlation, thus the Spearman coefficient of correlation has been preferred to the Pearson's one, since this appears to be more appropriate to describe the relationship between evapotranspiration and plant shoot system area.
